# A Statistically Supported Antioxidant Activity DFT Benchmark—The Effects of Hartree–Fock Exchange and Basis Set Selection on Accuracy and Resources Uptake

**DOI:** 10.3390/molecules26165058

**Published:** 2021-08-20

**Authors:** Maciej Spiegel, Andrzej Gamian, Zbigniew Sroka

**Affiliations:** 1 Department of Pharmacognosy and Herbal Medicines, Wroclaw Medical University, Borowska 211A, 50-556 Wroclaw, Poland; zbigniew.sroka@umed.wroc.pl; 2 Hirszfeld Institute of Immunology and Experimental Therapy, Polish Academy of Sciences, Rudolfa Weigla 12, 53-114 Wroclaw, Poland; andrzej.gamian@hirszfeld.pl

**Keywords:** density functional theory, polyphenols, computational chemistry, benchmark, caffeic acid, Hartree–Fock exchange, basis set, Janak’s theorem, theoretical chemistry

## Abstract

Polyphenolic compounds are now widely studied using computational chemistry approaches, the most popular of which is Density Functional Theory. To ease this process, it is critical to identify the optimal level of theory in terms of both accuracy and resource usage—a challenge we tackle in this study. Eleven DFT functionals with varied Hartree–Fock exchange values, both global and range-separated hybrids, were combined with 14 differently augmented basis sets to calculate the reactivity indices of caffeic acid, a phenolic acid representative, and compare them to experimental data or a high-level of theory outcome. Aside from the main course, a validation of the widely used Janak’s theorem in the establishment of vertical ionization potential and vertical electron affinity was evaluated. To investigate what influences the values of the properties under consideration, linear regression models were developed and thoroughly discussed. The results were utilized to compute the scores, which let us determine the best and worst combinations and make broad suggestions on the final option. The study demonstrates that M06–2X/6–311G(d,p) is the best fit for such research, and, curiously, it is not necessarily essential to include a diffuse function to produce satisfactory results.

## 1. Introduction

In recent years, dietary polyphenols have gained prominence as useful compounds due to their beneficial ability to scavenge reactive oxygen, nitrogen, and sulfur species and, in some cases, chelate transition-metal ions responsible for free radicals production [1,2,3]. The need for such investigations stems from the well-established detrimental impact of oxidative damage on body processes and the need to mitigate it [4]. Currently, a variety of methods are being used to study the antioxidant activity they display. Certainly, the most popular are laboratory assays that provide a quantitative description of the processes, allowing comparisons between compounds tested, but their key drawbacks is that they are mechanism-specific. For example, FRAP [5] or ABTS [6] measure electron transfer potential, while DPPH [7] evaluates hydrogen atom channel feasibility. Another major drawback is the absence of a qualitative aspect. To resolve these shortcomings, a study may be expanded or conducted exclusively using low-cost and satisfying quantum chemistry approaches that offer insight at the atomic level. The foundations are to estimate the intrinsic reactivity indices—bond dissociation enthalpy (BDE), ionization potential (*IP*), electron affinity (*EA*), and proton affinity (*PA*) [8]—which are viewed as a numerical definition of the reaction channels’ thermodynamic feasibility. It is also worth noting that kinetic studies of them are being carried out thoroughly and with pleasing precision [9].

BDE
$Antioxidant–OH\to Antioxidant–O^{•}+H^{•}$
IP
$Antioxidant–OH\to {\left[Antioxidant–OH\right]}^{•+}+e^{–}$
EA
$Antioxidant–OH+e^{–}\to {\left[Antioxidant–OH\right]}^{•–}$
PA
$Antioxidant–OH\to Antioxidant–O^{–}+H^{+}$


The electronic structure methods, collectively known as a Density Functional Theory, are the most widely used for that. The “DFT Zoo” has evolved over the years, and as a result, a diverse set of functionalities is now available [10]. The primary cause of this state is a relatively simple approach in the energy derivation method, which greatly reduces computations. Briefly, DFT obeys Hohenberg–Kohn’s first theorem [11], and hence, the ground state energy can be derived directly from the electron density distribution *ρ*, rather than from the many-electron wave function. The way of the calculating *ρ* value within the volume *r* involves just integration over the spin (*σ*) and *N* − *1* spatial coordinates (*x*) of probability density for all electrons (*N*) considered, as shown by the following formula (Equation (1)):

(1) $$\rho \left(r\right)=N\int \cdots \int {\left|\Psi \left(x_{1},x_{2},\ldots ,x_{N}\right)\right|}^{2}d\sigma_{1}dx_{2}\ldots dx_{N}.$$

Then, the system’s ground state energy becomes a function of density (*E[ρ]*) and is determined using the Kohn–Sham Equation [12], which fulfills the variational principle as follows (Equation (2)):

(2) $$E_{exact}\leq E\left[\rho \left(r\right)\right]=T\left[\rho \left(r\right)\right]+\int \rho \left(r\right)v\left(r\right)dr+E_{ee}.$$

It represents a hypothetical system of non-interacting electrons, with the first term being kinetic energy, the second denoting interactions with external potential (*ν*), and the third expressing electron–electron interactions. The last one can be further expanded (Equation (3)):

(3) $$E_{ee}=\frac{1}{2}\iint \frac{\rho \left(r\right)\rho \left(r\prime \right)}{\left|r–r\prime \right|}drdr\prime +E_{xc}\left[\rho \left(r\right)\right].$$

There, *E_xc_* is the sum of the exchange and correlation functionals that characterize non-classical electron–electron interactions and kinetics in the real system, and its existence is imposed by the antisymmetry and correlation requirements. During a self-consistent reaction field procedure, all terms are determined: all but the exchange–correlation term, which must be explicitly specified.

That leads to the five levels of accuracy that can be distinguished accordingly to “Jacob’s Ladder” proposed by Perdew [13]. The first one is represented by functionals establishing the electron density from the Local Density Approximation [11] (*LDA*), which has nearly become obsolete due to the significant simplification of asserting that the total exchange–correlation energy is the sum of local contributions, which furthermore are generalized to the uniform electron gas at the given density. The second and third rung functionals are, respectively, Generalized Gradient [14] (*GGA*), which takes into account the gradient of the electron density, and meta-GGA [15], which uses the second derivative instead. Finally, the penultimate rung is expressed by hybrid functionals [16], which have advanced significantly and are the most widely used DFT methods. Although the energy within them is calculated using LDA, GGA, or meta-GGA, regarding the functional used, the main distinction is the inclusion of an exchange–correlation term containing an arbitrary fraction of Hartree–Fock exact exchange (*%HF*). Two types of hybrid functionals can be distinguished: global hybrids (*GHs*), in which the value of the exchange interaction term is constant throughout the system, and range-separated hybrids (*RSHs*), in which the %HF varies based on the form of interaction, namely short or long. Sometimes, a middle range is separated as well, as in the case of HISSbPBE, which will be discussed within this paper.

There is no standard formulation of the exchange–correlation term, which leads to a wide variety of DFT functionals. Thus, the logical dilemma of whether to use it emerges. It is well understood that proper selection has a significant effect on the final results—while the given functional may yield the best kinetic data, it may also yield the worst description of excited states, and the aforementioned can be further amplified by the overall system structure [9,10,17]. That is why selecting the right method and basis set is critical, particularly in multi-step jobs where using an untested combination at the beginning could result in biased outcomes and, as a result, prejudice in the study’s conclusions [18]. This is especially true when there is a lack of comparable experimental evidence, requiring researchers to rely solely on theoretical findings. Furthermore, the margin of error is caused by the foundations—the approximations made in quantum mechanics—and can never be avoided. It must, though, be kept to a bare minimum.

Finally, the time and computational resources needed to execute computations with the desired precision are also relevant. Considering that HPCs are now readily available and that more sophisticated analyses can be done on them, using a high-end method when a much cheaper one can yield results with statistically negligible deviation from the aforementioned one makes little sense.

**Figure 1:**
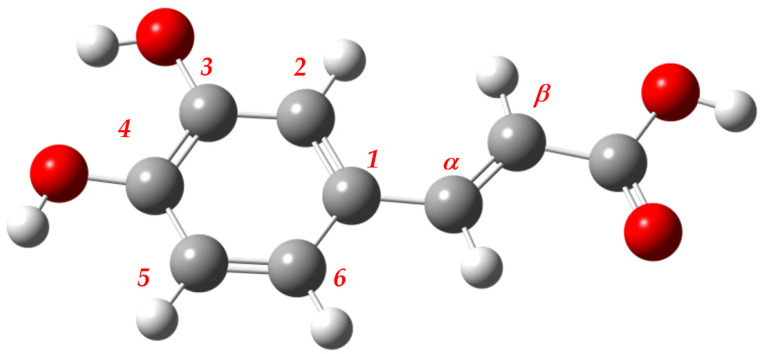
The 3D structure of the reference conformer of caffeic acid.

The aim of the research presented in this paper was to find the best method and basis set combinations for studies on the antioxidative and antiradical action of phytochemicals. Caffeic acid was chosen as a reference structure (Figure 1), and the logic for it is provided in the “Materials and Methods” section. This was achieved by investigating the geometry that underpins all and evaluating previously presented reactivity indices in relation to accessible experimental data and a high-level reference structure (consult Supplementary Materials). The importance of Hartree–Fock exact exchange fractions and “basis set”-linked features is discussed together with the simple linear regression models. The applicability of Janak’s theorem has been checked, as has the use of computing resources. Finally, the decision on the best and the worst combinations was made using the scoring function.

## 2. Results

The best and worst combinations were chosen based on our scoring function, which is presented in the “Materials and Methods” section. The estimated score is shown in Table 1.

The best functional, according to it, is M06–2X, which is supported by other studies that confirm its applicability and accuracy [19,20,21]. WB97 and M11 are two RSHs that are slightly worse, but they might also be considered in the studies. BLYP, MPWB1K, and HISSbPBE had the lowest overall results and thus are usually discouraged. Different constructions of Minnesota functionals have a significant impact on their performance, resulting in outcomes that are seldom comparable with the trends found for more typical DFT methods. In addition, it should come as no surprise that BLYP received the lowest score. Since it is the only functional that does not combine exchange energies calculated from Hartree–Fock with those obtained using DFT methods, it confirms that even a small amount of %HF is needed for accurate results.

In terms of basis sets, it appears that Pople’s triple-ζ is the most satisfactory, and, as stated in the preceding sections, it does not necessarily need to have a diffused function. Except for aug–cc–pVDZ, the lowest score was specifically associated with Dunning’s basis sets and Ahlrich’s def2-SVP and def2-TZVPD.

Finally, among all provided combinations, the studies have shown that the highest scores are obtained for M06–2X/6–311G(d,p), M06–2X/aug–cc–pVDZ, and M06-2X/cc–pVTZ, and thus, we generally recommend them for the studies, but we leave the final option to the researchers, depending on the purposes and resources available.

Calculations on bond dissociation enthalpies were performed on an extra set of compounds for which experimental values are available in order to better support the suggested level of theory for general investigations on polyphenols. The identical protocol as in the benchmark section was utilized. We choose to concentrate on the other group of polyphenols, flavonoids, to guarantee that our proposed method and basis set is applicable to them as well. The findings are shown in Table 2. As can be observed, the bulk of the calculated and experimental data discrepancies (Δ(*BDE_calc_* − *BDE_exp_*)) are less than ±4.0 kcal/mol—the best one was achieved for (–)-epicatechin, which varied from the known experimental data by just 0.4 kcal/mol. Catechin has the largest underestimation of BDE (−6.3 kcal/mol), whereas chrysin has the greatest overestimation (7.2 kcal/mol). The discrepancy between experimental and computational estimates for gallic acid, the only representative of phenolic acids from a distinct class, is just 2.0 kcal/mol. After all, the findings are satisfactory, with MAE = 3.8 kcal/mol and RMSE = 4.2 kcal/mol, especially given that totally different structures than those benchmarked were examined here.

## 3. Discussion

Prior to analyzing the computational outcome, we had to determine which hydroxyl groups the experimental values identified. For the time being, the experimental data suggest that if C4–OH is present, it would primarily undergo hydrogen-related channels. Furthermore, due to the much higher energy needed for bond cleavage, the participation of the carboxyl–OH group can be omitted. The reported bond dissociation enthalpies averaged out to 80.0 kcal/mol, which varied by 0.1 kcal/mol from the high-level theoretical result for C4. The gap in C3 was even greater, reaching 12.6 kcal/mol. We reproduced the method for proton affinity and discovered that the reference theoretical PA value of C4 differed from the observed experimental value by around –2.9 kcal/mol, compared to 17.0 kcal/mol for C3. We have already assumed that our reference level of theory accurately estimates energetics and therefore can be used satisfactorily for the remaining reactivity indices for which there are no literature values. A full list can be found in the Supplementary Materials.

### 3.1. Bond Dissociation Enthalpy

**Figure 2:**
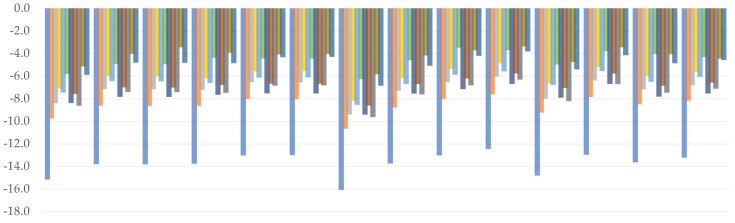

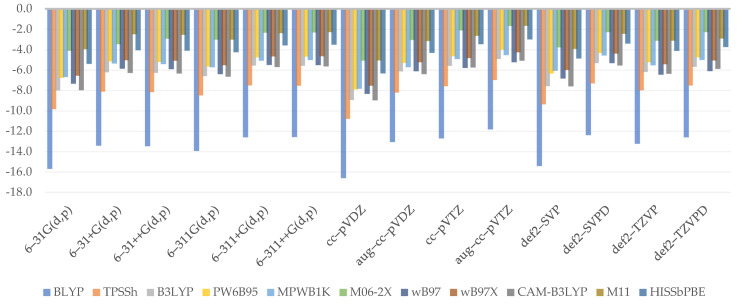
Relative errors to the reference value for C3 (**upper**) and C4 (**lower**) hydroxyl bond dissociation enthalpy. (kcal/mol).

All of the combinations generated underestimated values of bond dissociation enthalpies, as seen by the plotted charts (Figure 2). For both hydroxyl groups, Thrular’s M06–2X (*C3*: 4.6 kcal/mol; *C4*: 3.0 kcal/mol) and M11 (*C3*: 4.2 kcal/mol; *C4*: 3.0 kcal/mol) have been shown to provide the most reliable outcomes. BLYP should be labeled with the higher MAE value once more, since the yielded values are the worst of any examined functional (*C3*: 13.8 kcal/mol; *C4*: 13.6 kcal/mol). A trend may be identified within GHs at first glance—when we examine how an increase in %HF affects BDE values, we can see that it reduces the degree of underestimation, eventually approaching the comparison point. However, there is an exception to this rule in the case of PW6B95 and MPWB1K functionals, where the discrepancy is negligible despite a 10% difference. Importantly, regardless of the combination used, the lowest BDE was always associated with C4 hydroxyl group; a similar situation takes place in the case of proton affinity.

We related WB97 with WB97X to see how the short-range term affected them. Based on the comparison, it was discovered that while WB97X was associated with better results, they differed from those obtained by WB97 by just around 1.0 kcal/mol. Following that, and assuming that minor changes in the short-range term will have little effect on the final outcome, WB97X and CAM–B3LYP can be expected to vary only in the long term. For this pair, the latter was found to understate the result the most, placing it mostly below WB97. Based on this finding, we assume that long-range interactions are more important than short-range interactions, and that decreasing the value of the terms responsible for their representation is bounded by decreasing the BDE value. Examining the effect of the medium-range term, for example, by contrasting it to the functional of approximately equal value of the long-range term, as is the case for the HISSbPBE and CAM–B3LYP pair, we can see that the first one approaches the target much more smoothly, despite the fact that no other parameters are defined within it. As a consequence, we conclude that the medium-range term is the most significant, followed by the long-range and, finally, the short-range one.

We can also see from the predicted values that double-ζ basis sets are ineffective for calculating BDE because they tend to sharply underestimate the values of this reactivity index. The cc–pVDZ produced noticeably weak results (*C3*: 9.1 kcal/mol; *C4*: 8.5 kcal/mol), but transitioning to the diffused triple-ζ inside the same basis set family, namely aug–cc–pVTZ, yielded the best outcomes (*C3*: 6.0 kcal/mol; *C4*: 4.9 kcal/mol). While the influence of the mentioned modification is noticeable in either Pople’s, Ahlrich’s, or Dunning’s basis sets, it is most prominent in the latter one. In general, we can see that diffused double-ζ basis sets generated values similar to non-diffused triple-ζ basis sets of the same family. This property may be important in the sense of calculation performance, as diffusion is known to take significantly more computational resources.

(4) $$\begin{array}{l} Y_{BDE\_C3}=1.747\times 10^{-2}{SR}^{*}+8.320\times 10^{-2}{MR}^{***}+4.429\times 10^{-2}{LR}^{***}\\ \;+2.721\times 10^{-3}{NBF}^{**}+5.351\times 10^{-1}D^{*}-1.249\times 10^{1}{}^{***} \end{array}$$

(5) $$Y_{BDE\_C4}=0.102{MR}^{***}+0.061{LR}^{***}-1.003\zeta^{***}+1.219D^{***}-11.182^{***}.$$

The coefficients obtained from our two regression models (Equations (4) and (5)) indicate that the medium- and long-range terms play an important role in estimating bond dissociation enthalpy for both C3 and C4 hydroxyl groups. This adds to the conclusions and debate provided in the preceding paragraphs. Furthermore, the observed similarity between diffused double-ζ and undiffused triple-ζ was reflected in the C4 model—in the first example, two terms are present, creating a slight increase in the total value (1.219 − 1.003 = 0.216), while in the second, both are missing, leaving the estimate of the BDE value to intercept, as well as MR and LR coefficients. Surprisingly, in the case of C3, the position of the diffuse feature is on the very edge of statistical significance. In both models, the intercept has a high statistical significance and the greatest, though negative, value. This means that some essential elements are missing from our basic models and should be reconsidered. One of them may be resonance stabilization energy, which is particularly significant in the case of C4, where delocalization extends into the side chain after bond cleavage due to orbitals’ conjugation. Other possibilities include hydroxyl oxygen repulsions and the formation of hydrogen bonds during the reaction. The latter one is less important for C3 bond cleavage because forming a hydrogen bond would necessitate rotation of the C4 hydroxyl hydrogen and additional work. Nonetheless, they are represented by the satisfactory *R*^2^ values of 0.6775 and 0.7384, and residual errors of 1.519 and 1.532 for C3 and C4, respectively. Furthermore, the obtained *F*-statistic indicates that they are valuable.

### 3.2. Adiabatic Ionization Potential

**Figure 3:**
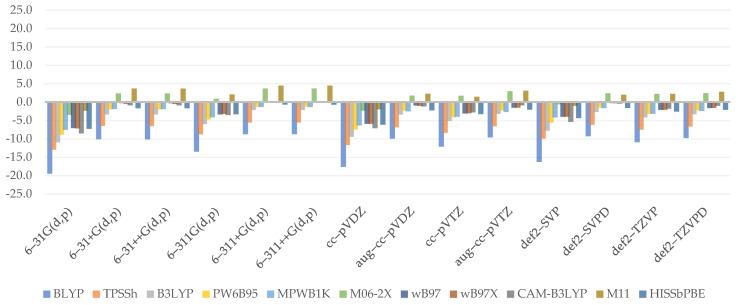
Relative errors to reference value for adiabatic ionization potential (kcal/mol).

There was no mention of adiabatic ionization energies in the literature. As a result, we compared our findings to the result of our reference level of theory, B2PLYP–D3/aug–cc–pVTZ, which equals 184.1 kcal/mol. As with bond dissociation energy, the findings (Figure 3) were nearest to the reference value for Minnesota’s functionals M06–2X (2.4 kcal/mol) and M11 (2.7 kcal/mol)—these are the only functionals that overestimate the outcome regardless of the basis set used. Aside from them, some popular RSHs performed well, including wB97 (2.3 kcal/mol), wB97X (2.3 kcal/mol), and CAM–B3LYP (2.5 kcal/mol). Not surprisingly, BLYP was once again the worst choice (11.9 kcal/mol).

In the case of GHs, the effect of increasing %HF is noticeable as a proclivity to approach the reference value, with M06–2X being the closest. However, when comparing range separation schemes, it is difficult to discuss the position of the short-range term because WB97, WB97X, and CAM–B3LYP produce comparable results, while M11 differs and produces the best. A similar logic to that given in the paragraph devoted to hydrogen bonds length may be at work. As a result, we believe that neither the short- nor long-range terms had a significant effect, at least among GGA functionals, but that the short-range term could have double the impact of the long-range term. The results of HISSbPBE are also difficult to interpret, especially because they are similar to those obtained by WB97 or WB97X. That is, the influence of the medium-range term should be twice as strong as the impact of the long-range term. As we move on to the topic of basis sets, the picture begins to clear up. In general, it appears that the presence of the diffuse function is supposed to correctly predict ionization potential. Furthermore, double-ζ basis sets have been shown to yield significantly underestimated values, which can be easily corrected by switching to triple-ζ basis sets or, more ideally, simply augmenting it with a diffuse function, resulting in a major correction in the given adiabatic ionization potential value. Lastly, the augmented version produces results that are closer to the reference value than the undiffused triple-ζ, pinpointing the role of diffusion. In the case of Pople’s basis sets, this encloses just in its presence, regardless of whether a single or double one is used. As a result, the most satisfactory findings came from 6–311+G(d,p) (2.6 kcal/mol), 6–311++G(d,p) (2.6 kcal/mol), and Ahlrich’s def2–SVPD (2.6 kcal/mol).

(6) $$Y_{aIE}=0.031{SR}^{**}+0.100{MR}^{***}+0.087{LR}^{***}-1.696\zeta^{***}+3.605D^{***}-11.393^{***}$$

All features except the basis set size are statistically significant, according to the established regression model (Equation (6)). To begin with, we can see that increasing the Hartree–Fock exchange term at any range causes an increase in the expected adiabatic ionization potential, which in our series of combinations is usually associated with improved outcomes and their enclosure within the X-axis. Furthermore, the coefficients for the medium and long range are identical but greater than the one for short range. Actually, the coefficient of the short-range term is the lowest, and a 20% HF discrepancy between two functionals will result in an energetic disparity of just 0.6 kcal/mol. That may explain why WB97 and WB97X are so similar to each other. A similar treatment for WB97X and CAM–B3LYP yields a 3.0 kcal/mol gap, but this one is not apparent. According to the model we presented here, its *R*^2^ is 0.8147, *F*-statistics indicate that it is statistically significant, and the residual standard error varies around 1.924.

### 3.3. Adiabatic Electron Affinity 

**Figure 4:**
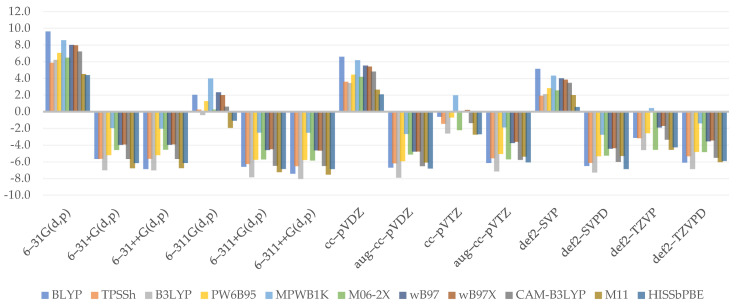
Relative errors to reference value for adiabatic electron affinity (kcal/mol).

The reliability of electron affinity computed at lower levels was determined by comparing it to the reference values, which equals –12.8 kcal/mol. The plotted data (Figure 4) reveal that extrema are far closer to the 0 value than in the case of adiabatic ionization potential, implying that DFT methods estimate adiabatic electron affinity better. Overall, the effects are somewhat stable, as shown by the MAE values, the lowest of which was expressed by MPWB1K (3.1 kcal/mol) and the highest by BLYP (5.7 kcal/mol) and B3LYP (5.6 kcal/mol).

Aside from the BLYP, GHs usually provide higher values as the %HF increases. B3LYP has been found to defy this law, especially when diffused, or generally, larger basis sets are combined with it. As can be shown, WB97 and WB97X render adiabatic electron affinity values almost at the same grade, implying that the short-term effect is insignificant. However, as CAM–B3LYP is analyzed, the values noticeably decrease. That, on the one hand, provides a basis for the presumption of the effect provided by the long-range term, which is not inherently significant but is greater than the short-range term, in which no major variations were detected. M11 distinguishes itself once more by producing even lower values than expected. The importance of the medium range, which was clearly shown by the projected outcomes when HISSbPBE was used, is uncertain. The findings of diffused, Dunning’s, or Ahlrich’s basis sets are similar to those of CAM–B3LYP. When no such basis set is used, the difference between them will exceed 4 kcal/mol (in the case of the smallest basis set, 6–31G(d,p)). The inconsistencies discovered in this paragraph provide clear evidence that similarly to adiabatic ionization potential, a basis set can have the greatest effect on overall results.

To begin, the chart shows that in the case of electron affinity, the choice of double-ζ is often insufficient, resulting in vastly exaggerated results. On the other hand, augmenting with diffusion causes the findings to be significantly understated in each situation. The golden mean happens to be the recruiting of the undiffused triple-ζ, which tends to provide the best results; however, this does not apply to Ahlrich’s def2–. As a result, it is not surprising that 6–311(d,p) (1.7 kcal/mol) and cc–pVTZ (1.5 kcal/mol) are representative basis sets for adiabatic electron affinity computations. As most basis sets provide unsatisfactory results, the greatest MAE is correlated with 6–31G(d,p) (6.9 kcal/mol).

(7) $$Y_{aEA}=0.012{LR}^{**}+2.416\zeta^{***}-7.203D^{***}$$

The obtained regression equation for adiabatic electron affinity (Equation (7)) is the simplest of all those found in the paper. It is also among the best, with an *R^2^* of 0.7788, passing the *F*-statistics criteria, and a residual standard error of 2.394. It states that the outcomes of adiabatic electron affinity can be calculated using only the long-range term and taking into account the type of basis set used as well as the presence of a diffuse function. The relative effect, as assigned to coefficient values, is in reverse order. Since range separation schemes play no part, this explains why we were unable to find a viable pattern. The role of the last two coefficients, on the other hand, has already been narrowly explained in the preceding paragraph. Indeed, the linear model confirms how the diffuse function contributes significantly to the final outcome and how this effect is underestimated when the basis set is changed from double to triple.

### 3.4. Proton Affinity

**Figure 5:**
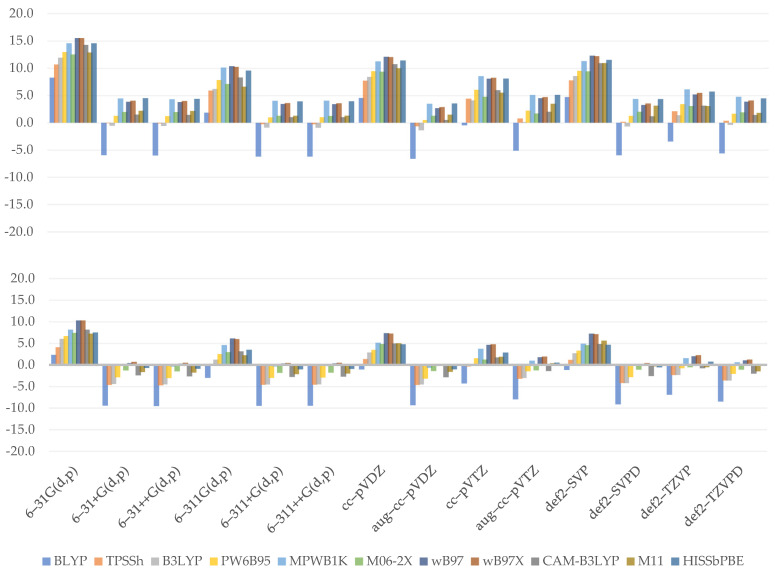
Relative errors to reference value for C3 (**upper**) and C4 (**lower**) hydroxyl proton affinity (kcal/mol).

Similar to bond dissociation, proton detachment may occur at any available hydroxyl hydrogen. At first sight, the plotted charts (Figure 5) do not seem to be those of the bond dissociation enthalpy. The values obtained for proton affinity at C4 tend to be estimated with greater accuracy than those obtained for C3, as shown by the MAE values: TPSSh was discovered to have the most reliable result for C3 (3.0 kcal/mol). HISSbPBE (*C3*: 6.8 kcal/mol*; C4*: 2.2 kcal/mol) and MPWB1K (*C3*: 6.9 kcal/mol*; C4*: 2.3 kcal/mol) produced satisfactory results for this deprotonation site too, though the worst for the C4 location. WB97X reported similarly poor results for C3 (6.7 kcal/mol) and BLYP produced poor results for C4 (6.6 kcal/mol).

The figure exhibits a pattern showing that an increase in Hartree–Fock exchange causes an increase in results found for GHs. WB97 and WB97X vary only in a short range and tend to yield comparable results, while M11 reported more reliable results once again. Assuming, once again, that the short-range influence is marginal as compared to others, the decline in a long range, as seen in the contrast of WB97X and CAM–B3LYP, seems to be responsible for the lower outcome. HISSbPBE has been observed to produce results that vary between those produced by WB97 or WB97X and CAM–B3LYP, implying that there is no substantial effect of the medium-range term and that it may be mathematically evaluated as around twice the long-range term influence, which could be true if we note that CAM–B3LYP yields approximately the same results, with a 15% greater exact exchange at the long-range term. The findings further show that the basis set used might have a larger impact on the results; namely, the best were found for augmented ones. This is due to the observation that as the percentage of HF exchange increases, only the diffused basis sets approach the reference value.

When we examine the effect of the basis set selection, we can see that every diffused basis set, whether double- or triple-ζ, is a good one. Dunning’s aug–cc–pVTZ is an exception, with results that are slightly weaker than those achieved by the non-augmented cc–pVTZ. After all, the majority of them accurately estimate the desired values, and only 6–31G(d,p) is associated with significant MAE (*C3*: 13.1 kcal/mol; *C4*: 7.1 kcal/mol).

(8) $$Y_{PA\_C3}=-0.053{SR}^{***}+0.101{MR}^{***}+0.071{LR}^{***}+2.099\zeta^{***}-6.699D^{***}+2.825^{***}\;$$

(9) $$Y_{PA\_C4}=-0.049{SR}^{***}+0.092{MR}^{***}+0.078{LR}^{***}+1.377\zeta^{***}-5.349D^{***}-2.100^{***}$$

As seen by the equations of the developed regression models (Equations (8) and (9)), only the short-range term is responsible for the decrease in enthalpy value and can be easily resolved by the coefficients of medium- and long-range terms. On the other hand, the negative impact of long range posed for WB97X and CAM–B3LYP proton affinity is not confirmed. Similarly, the impact of a medium-range term is not the same as what is observed. Models find it much simpler to estimate the role of basis sets—indeed, double-ζ basis sets induce an increase in process energy, although a smaller one than that observed. The effect of diffusion is also evident, but it tends to be overlooked within a model in the same way as the influence of base set type is. Overall, it provides a clear qualitative analysis of how each function impacts the final outcome, as shown by *R^2^* for C3 equaling 0.7956 and *R^2^* for C4 equaling 0.7621. Residual errors are 1.882 and 2.37, respectively, and all pass the statistical significance threshold as measured by *F*-statistics. Finally, the intercept is statistically significant in both of these models.

### 3.5. Section Conclusions

We attempted to go into detail in this section on how different basis sets and functionals affect the estimates of specific antiradical activity indices. To support our theoretical findings, we established a variety of linear models, the majority of which had a satisfactory coefficient of determination. Contrary to popular opinion, the use of a diffuse feature is not necessarily needed and can be sufficiently substituted in certain cases by a triple-ζ. Furthermore, we defined the role of RSHs in such studies and provided insight into the Ahlrich’s and Dunning’s basis set families, indicating that they should be considered in such studies.

### 3.6. Performance Evaluation

**Figure 6:**
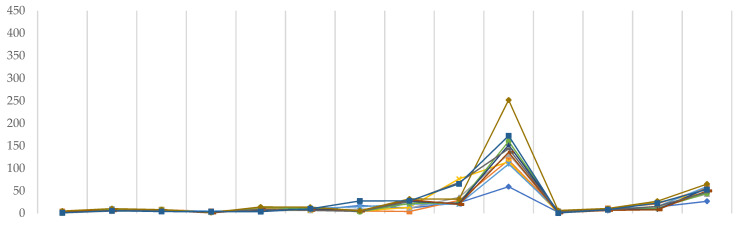

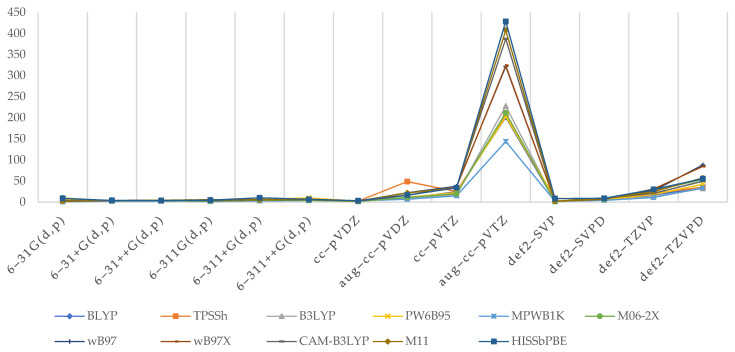
Average time required to complete a single instance of Link502 (**upper**) and Link703 (**lower**) executables using a single CPU (s).

All of the points of the graph (Figure 6) have been standardized to reflect the average time taken to complete one instance of *Link502* or *Link703* executables when only one CPU is used. Since random access memory has only a minor impact on the pace of DFT calculations, it was not taken into account in this study.

As can be shown, functionals have little effect on efficiency; however, the lines in *Link502* are not as well overlaid as in *Link703*, indicating that iterative SCF calculations are more vulnerable to functionals selection. There, we can see that discrepancies are beginning to emerge for Ahlrich’s def2–TZVP and def2–TZVPD families, as well as for Dunning’s correlation-consistent family. M11 tends to take the most CPU time in each of these scenarios. Furthermore, in the case of *Link703*, and only there, it was discovered that TPSSh/aug–cc–pVDZ had nearly three times the computational time of any other combination there.

The selection of the basis set has a much larger impact, since the number of base functions necessarily corresponds to the greater number of calculations to be completed, and hence, they are more vulnerable to available computing resources. There are no major variations in Pople’s family, though the SCF procedure seems to be somewhat longer in the case of diffused triple-ζ basis sets. While Ahlrich’s basis sets exhibit a similar trend, def2–TZVP and def2–TZVPD need significantly more time than 6–31G or 6–311G. Ultimately, in the case of correlation-consistent basis sets, the computing resources required to complete each *Link* start to vary greatly. In their case, first, greater fluctuations occur at cc–pVDZ of *Link502* and reach a maximum for aug–cc–pVTZ, the largest and most resource demanding basis set—in both SCF method and two-electron integral calculations.

### 3.7. Janak’s Theorem Applicability

**Figure 7:**
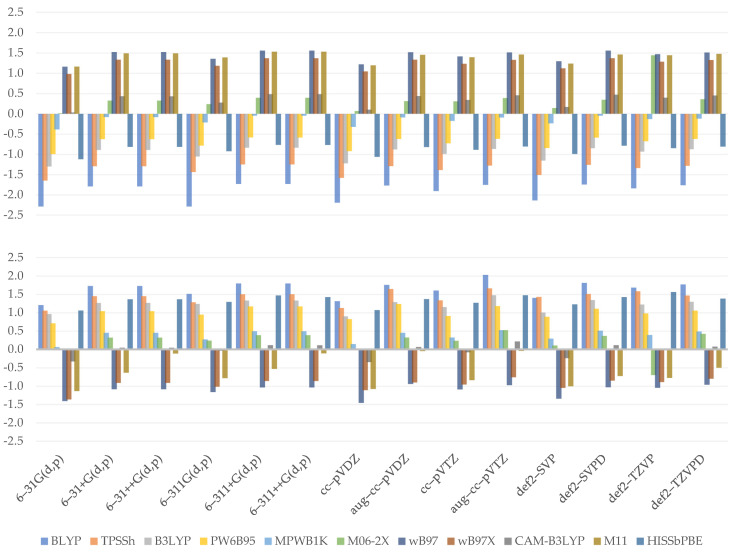
Calculated errors to reference value for vertical ionization potential (**upper**) and vertical electron affinity (**lower**) using Janak’s theorem (eV).

As previously said, approximating orbital energies is a quite common method for estimating vertical ionization potential (*vIP*) and vertical electron affinity (*vEA*). As seen in the figures above (Figure 7), the errors vary considerably, ranging from 1.5 to –2.3 eV. Since 1.0 eV equals approximately 23.0 kcal/mol, the disparities are significant; therefore, the benefit of the doubt in choice is at least not recommended. The values were compared to the reference molecule’s (errors: *vIP =* 7.2 eV and *vEA =* 0.7 eV). Of all functionals, BLYP produced the worst results (errors: *vIP =* 1.9 eV and *vEA =* 1.7 eV), while MPWB1K produced the best vIP (error: 0.2 eV) and CAM–B3LYP produced the best vEA (error: 0.1 eV).

When we look at the significance of the Hartree–Fock exact exchange in the context of GHs, we can see that increasing its value results in an increase in the values obtained for vIP and a decrease in the values obtained for vEA. After all, this brings us closer to the reference value. The role of the short-range term, as expressed by the difference between WB97 (which represents the maximum or minimum, respectively, for vIP and vEA), WB97X, and M11, suggests that this feature is of minor importance. As can be shown, increasing the short-range term has little effect on the vIP, while in the case of vEA, the outcomes are slowly approaching zero. However, in case of the EA, a greater impact is visible for M11, particularly when combined with Pople’s or Dunning’s augmented basis to predict vertical electron affinity. With this in mind, we should say that the 5% difference in short range between CAM–B3LYP and WB97X is marginal to the results, and we can analyze the effect of the decrease in long range. It has been observed that the former produces significantly more accurate results, especially for the vEA. According to the data, decreasing the long-range term value seems to result in a much closer match to the desired outcome, implying that it is essential for proper approximation. Finally, HISSbPBE, a representative functional with only a medium-range term, is found to yield results at the degree of TPSSh or B3LYP. When studying HISSbPBE, we reuse previously introduced assertions that an increase in short range leads to steadily approaching the reference value, and a decrease in long range does the same but faster; we assume that a medium-range term has twice as much effect as a long-range term.

In terms of basis sets, it is worth noting that even the simplest one can be used, as shown by the MAE of vEA, the lowest value of which was observed for 6-31G(d,p) (0.8 eV). On the other hand, aug–cc–pVDZ has the lowest MAE of vIP (1.0 eV). In the case of vEA, the greatest MAE is observed for aug–cc–pVTZ (1.0 eV) and def2–SVPD (1.0 eV), and def2–TZVP for both vIP (1.1 eV) and vEA (1.0 eV). Looking at the chart, we can see that switching from a double- to a triple-ζ causes a rise in both values. When diffusion is introduced, the effect is similar, but when applied to double-ζ, the effect is greater than when applied to triple-ζ.

(10) $$Y_{vIP}=-0.006{SR}^{***}+0.014{MR}^{***}+0.033{LR}^{***}-0.108\zeta^{***}+0.192D^{***}-1.860^{***}$$

(11) $$Y_{vEA}=0.008{SR}^{***}-0.007{MR}^{***}-0.028{LR}^{***}+0.283D^{***}+1.601^{***}$$

Based on our results, we generated linear regression models, attempting to correlate proposed features in a linear model. According to statistics, the *R^2^* of the models is 0.9805 in the case of vIP (Equation (10)) and 0.9755 in the case of vEA (Equation (11)), indicating that they are really fine. Both have a residual error of around 0.1570, and statistical validity is confirmed using *F*-statistics. As one can see, the coefficients describing separation schemes are mirror images, with LR being the greatest one. Although the task of double-/triple-ζ appears to be important for the vIP model, no such thing was found for vEA.

To summarize this part, Janak’s theorem is valid to correctly predict the vertical ionization potential and vertical electron affinity, but the results are very susceptible to changes. As a consequence, using this approximation should be judged and avoided unless M06–2X or M11 functionals are used.

## 4. Materials and Methods 

### 4.1. Caffeic Acid as a Reference Structure

Caffeic acid is one of the most basic and readily available plant antioxidants found in the diet [23,24,25]. Its chemical structure contains all of the features needed for effective scavenging of free radicals, namely conjugated double bonds and hydroxyl hydrogens, which form a catechol moiety in this case. Its presence is well known to be a determinant of high antiradical activity and chelating properties [26,27]. In the scope of the paper, the benchmark results obtained for the small compound can be easily applied in the larger system.

In general, it would be better for the analysis to focus on the global minimum conformer; but, using computational chemistry techniques, this is almost impossible. To some extent, the X-ray structures can be representative, but the geometry deposited cannot be guaranteed as a minimum due to deformations during crystallization caused by factors such as temperature and milieu; further optimizations at the desired level of theory are needed. As a result, we agreed to concentrate solely on computational data and produced a series of initial conformers through a molecular dynamics simulation [28]. At that time, we bypassed *cis*-conformers in favor of *trans*-, which are the least energetic [27,29]. The geometry with the lowest energy has been used as a starting point for all future DFT calculations.

### 4.2. On Functionals and Basis Set Choice

Eleven DFT functionals (Table 3) were selected and paired with 12 double- or triple-ζ basis sets (Table 4), yielding a total of 132 ground state conformers to investigate.

The global hybrids were chosen so that they span the spectrum of %HF from 0 to 50% and enclose in a near interval, providing a pattern of how the properties analyzed change with its increment. A similar approach was attempted for range-separated functionals in order to investigate the effect of exact exchange at short, middle, and long-range interactions. In a paper published a few years earlier, La Rocca et al. [19] chose functionals based on a similar condition, but it was done for edaravone and quercetin and with only four of Pople’s basis sets.

Balancing precision versus computational time is a critical component of any computational challenge, not just benchmark studies. Since the number of basis functions scales with the time required to complete the computations, it is generally preferable to choose the smallest but still precise one whenever possible. To begin, we completely avoided those that lacked polarization functions. Their position is clear: accurate representation of the strongly polarized O–H bond (χ =1.24) is crucial for studying hydroxyl bond length, bond break enthalpies, and atomic orbitals representation. Furthermore, they have no discernible effect on computational power use. Far more relevant are diffuse functions, which are known to slow down calculations but are thought to be necessary for proper studies on compounds with large electron clouds, particularly ions and radicals. In addition, the existence and role of hydrogen bonds within polyphenols encourages their use [8,41,42].

One would wonder why such unusual basis sets as Ahlrich’s or Dunning’s, or methods such as TPSSh or WB97, were chosen when Pople’s [53,54,55], particularly when combined with Minnesota’s functionals [26,56,57,58,59], are leading the way? At the time of writing this paper, we were barely able to find articles that used def2– [60] or (aug–)cc–pVXZ [61,62,63] basis sets in their studies on antioxidative activity. A similar situation occurred in the case of functionals where, with a few exceptions, the bulk of papers used B3LYP and Minnesota functionals. This is not to suggest they are untrustworthy, particularly in the case of Minnesota, where theoretical outcomes precision has been verified by developer-independent scientists [64,65,66]. Instead, we must accept that Pople’s basis sets and B3LYP were the first to be widely and successively adopted, resulting in their extensive use and accuracy tests “within” studies. While the reliability of the analyzed basis sets and functionals has not yet been checked for polyphenolic compounds, their value can be comparable to or even greater than that previously described. For example, bond and non-bond interactions have been successfully estimated using def2- and correlation consistent basis sets [10], and moreover, the balance between accuracy and calculations cost is appealing [67,68]. Similarly, when it came to determining ionization potential and electron affinity, which are both essential for some antiradical activity channels, WB97X outperformed other functionals studied [69]. We considered these to be valid justifications for incorporating them in our paper.

### 4.3. DFT Calculations

The coordinates of all geometries presented can be found in the Supplementary Materials. All but the reference level of theory calculations were performed in the Gaussian16 [70] quantum chemistry package, with a very tight geometry optimization cut-off and ultrafine integration grid, as is recommended for all DFT calculations. A vibrational analysis was conducted at each stage of the study to verify the absence of imaginary frequencies and to obtain enthalpy values. To get the ground state conformer, the previously described molecular dynamics geometry was optimized in each functional/basis set pair. Then, the structures obtained were used as templates to generate the radicals, ion radicals, and ions needed to determine the reactivity indices at the same level of theory. The findings were compared to the reference system results or, if available, experimental data.

We were able to find experimental evidence for BDE: 78.7 kcal/mol [71] and 81.2 kcal/mol [22] (averaged to 80.0 kcal/mol); similarly, for PA: 323.3 (±2.2) kcal/mol [72] through studying the literature. Since these values were obtained for the gaseous state, the computations were done in vacuum. This has allowed us to exclude several solvent effects from the linear regression models. To measure relative error to the reference value (*ε_f,b_*_,_ Equation (12)), the following formula was used:

(12) $$\varepsilon_{f,b}=X_{f,b}-X_{ref}$$

where *X_ref_* is the reference value of the property examined, and *X_f,b_* is the value of the property calculated in functional *f* and basis set *b*. Regarding the fact that it parallels the approximation error, we opted to ignore the modulus in order to keep track of whether underestimation or overestimation occurred. Unless some of the displayed results were particularly noteworthy, we generally discussed only mean absolute error (*MAE*) in a specific functional ($\varepsilon_{f}$, Equation (13)) or basis set ($\varepsilon_{b}$, Equation (14)):

(13) $$\varepsilon_{f}=\frac{1}{N_{b}}\overset{N}{\underset{b}{\sum }}\left(\left|X_{f,b}-X_{ref}\right|\right)$$

(14) $$\varepsilon_{b}=\frac{1}{N_{f}}\overset{N}{\underset{f}{\sum }}\left(\left|X_{f,b}-X_{ref}\right|\right)$$

where *N_f_* is the number of functionals and *N_b_* is the number of basis sets.

For reference purposes, the molecular dynamics geometry has been optimized using RI–MP2/aug–cc–pVTZ [50], along with the aug–cc–pVTZ/C auxiliary basis set [73], which are all implemented in ORCA [74]. Then, the energy corrections were performed using Grimme’s double hybrid functional RI–B2PLYP [75,76] with D3BJ dispersion corrections [77] in the same basis sets. Additionally, the exchange integrals calculations were sped up using the COSX algorithm [78,79,80]. BDE and PA have been computed at this level of theory to corroborate the choice of B2PLYP–D3BJ/aug–cc–pVTZ energies for computing the reference reactivity indices. When compared to the previously given experimental results, the errors were determined to be 0.08 kcal/mol and 3 kcal/mol, respectively.

### 4.4. Linear Regression Models

Linear regression models were developed to express mathematically how certain features of functionals and basis sets affect the results obtained for each property studied. These models provide a numerical representation of the degree of influence, its type, as well as insight into patterns that are not so readily observable. While we believe it is the first time they are used for the benchmark study, their role is well known from QSAR models [26,53,54] that enable evaluation of the effect of thousands of descriptors and fingerprints on the activity exhibited. All of the models were developed using the R programming language [81], and the following features were included within them:

- %HF at short range (*SR*, [0, 100]);
- %HF at middle range (*MR*, [0, 100]);
- %HF at long range (*LR*, [0, 100]);
- Number of basis functions (*NBF*, N > 0);
- Presence of valence double basis set (*ζ,* 0 ∨ 1);

*Note: The absence of double-ζ is automatically viewed as an inclusion of triple-ζ.*

- presence of diffuse function (*D*, 0 ∨ 1).

*Note: Due to the slight variation in findings between single and double diffusion, as well as the lack of such divergence in Dunning’s and Ahlrich’s basis sets, no distinction has been made between them.*

The models were optimized by excluding statistically insignificant coefficients. We decided to keep the intercept if it was statistically significant, so we could see how factors not actually used in our models influenced the result.

### 4.5. Computational Performance

To address computational performance, we have gathered CPU times and number of initializations of two main bottlenecks—iterative solution of SCF Equations (*Link502*) and two-electron integral first or second derivatives for s, p, d, and f orbitals (*Link703*)—for each method and basis set combination. An average CPU time/program instance for each of these two procedures was established and then normalized to the one core. We decided to make such an approximation, since according to output files we have been working with, it is burdened with a marginal error of 1–2 s.

### 4.6. Janak’s Theorem Revisited

Aside from adiabatic ionization potential and adiabatic electron affinity, it is sometimes necessary to estimate vertical ionization potential (*vIP*) and vertical electron affinity (*vEA*), e.g., when comparing antioxidative activity by using a donator–acceptor map [82,83,84]. The distinction is that in adiabatic geometry, relaxation after electron acceptance or donation is permitted, reducing the total energetics of the process. In the case of vertical variants, this is prohibited, and the geometry remains constrained.

Although the direct calculations are simple, these values are also believed to be conveniently obtained from the energy of the orbitals. According to Janak’s theorem [85], the DFT counterpart of Koopmans’ theorem, the vertical ionization potential, and the vertical electron affinity can be approximated by the negative eigenvalues of the HOMO and LUMO orbitals, respectively. Nonetheless, the DFT exchange–correlation energy approximation is known to cause a self-interaction error due to the residual interactions of the electron with itself, resulting in a contamination of the Kohn–Sham orbitals, as shown for example in a tendency to underestimate ionization potential energies [86]. Owing to that, the above indirect approach should be used with caution, especially in the case of GHs. Despite these challenges, according to recent comments, RSH functionals can solve them by providing results that are more reliable than those obtained by global hybrids [69]. To answer the method’s applicability, we checked whether any of the combinations yielded outcomes close to those obtained from the reference level of theory—vIP_ref_ = 7.16 eV and vEA_ref_ = 0.67 eV.

### 4.7. Scoring Function

To complete our paper and choose the best combination from our set, we used a scoring function (Equation (15)) to determine the success of each of them, where *S* represents the number of points assigned. The indices are as follows—*p* stands for the property studied, namely:

- Hydroxyl bond length at C3;
- Hydroxyl bond length at C4;
- Hydrogen bond length;
- Bond dissociation enthalpy at C3;
- Bond dissociation enthalpy at C4;
- Adiabatic electron affinity;
- Adiabatic ionization potential;
- Proton affinity at C3;
- Proton affinity at C4.

*cp* is the computational performance established in *Link502* or *Link703*; *J* represents a vertical ionization potential or vertical electron affinity computed according to Janak’s theorem; ξ stands for the value yielded by examined combination in each of the previously stated; and *X_ref_* remains as previously stated. For clarity, the score achieved by the specific level of theory is related to the formula’s maximum and expressed as a percentage.

(15) $$\begin{array}{l} SCORE=0.6\underset{p}{\sum }S_{p}+0.3\underset{cp}{\sum }S_{cp}+0.1\underset{J}{\sum }S_{J}\\ S_{J}=S_{p}=\{\begin{array}{c} 2\;if\;\left|\xi \right|<0.01\times \left|X_{ref}\right|\\ 1\;if\;0.01\times \left|X_{ref}\right|<\left|\xi \right|<0.05\times \left|X_{ref}\right|\\ 0\;if\;0.05\times \left|X_{ref}\right|<\left|\xi \right| \end{array}\\ S_{cp}=\{\begin{array}{c} 2\;if\;\varepsilon_{p}<0.01\times \max\left(\xi \right)\\ 1\;if\;0.01\times \max\xi <\xi <0.05\times \max\left(\xi \right)\\ 0\;if\;0.05\times \max\left(\xi \right)<\xi \end{array} \end{array}$$

## 5. Conclusions

According to our literature study, it is the very first such comprehensive, in terms of functionals and basis sets used, benchmark performed to determine the best method/basis set combination for polyphenol studies.

We were able to pinpoint the best and the worst one for each parameter analyzed by examining the most important geometrical features as well as reactivity indices, and finally estimate general performance, which also included Janak’s theorem applicability and computational resource use. Remarkably significant in terms of the last one was determining that although the diffuse functions had a mostly significant effect, they are not always needed and can be satisfactorily skipped. After all, M06–2X/6–311G(d,p) appears to be the very best choice for the calculations. From that point of awareness of its accuracy, the new possibilities in the thermochemical benchmarks on polyphenols opens, for example, the impact of solvation models.

The mathematical representation of the results obtained has given insight into what characteristics are critical in determining properties related to polyphenols’ antioxidant activity. About the fact that limited models were used, they often satisfied and validated observed trends. The observed differences between C3 and C4 hydroxyl groups are due to structural characteristics that have been thoroughly studied by other authors. To name a few, these include the ability to create a hydroquinone-like structure and extended delocalization in case of C4 hydrogen detachment, as well as mutual interaction between them [53,54,87,88].

We hope this approach prompts other researchers to recruit linear regression models, where more components e.g., the effect of the electron density approximation approach or the influence of the solvation method, are incorporated. However, most importantly, we expect that with this paper, scientists will find it easier to research polyphenols and investigate how they can protect us from the presence of oxidative stress.

## Figures and Tables

**Table 1:** The final score of each combination studied ^i^.

	BLYP	TPSSh	B3LYP	PW6B95	MPWB1K	M06-2X	wB97	wB97X	CAM–B3LYP	M11	HISSbPBE
**6–31G(d,p)**	41%	41%	46%	46%	41%	54%	53%	51%	55%	51%	46%
**6–31+G(d,p)**	32%	56%	51%	61%	57%	66%	61%	61%	65%	70%	60%
**6–31++G(d,p)**	32%	56%	51%	61%	57%	66%	61%	61%	63%	70%	60%
**6–311G(d,p)**	56%	61%	60%	56%	37%	78%	51%	51%	61%	65%	39%
**6–311+G(d,p)**	46%	53%	51%	63%	52%	65%	58%	58%	63%	65%	58%
**6–311++G(d,p)**	46%	53%	48%	63%	52%	70%	63%	58%	63%	65%	58%
**cc–pVDZ**	39%	46%	51%	46%	44%	53%	46%	46%	47%	46%	44%
**aug–cc–pVDZ**	29%	51%	48%	56%	42%	71%	53%	56%	61%	63%	51%
**cc–pVTZ**	44%	48%	46%	48%	32%	66%	53%	48%	49%	63%	48%
**aug–cc–pVTZ**	34%	48%	48%	53%	40%	63%	53%	53%	58%	58%	48%
**def2–SVP**	41%	46%	56%	46%	47%	61%	46%	53%	47%	56%	51%
**def2–SVPD**	39%	53%	48%	63%	52%	69%	58%	53%	63%	68%	68%
**def2–TZVP**	32%	56%	51%	56%	47%	65%	51%	51%	56%	63%	48%
**def2–TZVPD**	29%	48%	44%	53%	40%	64%	53%	53%	58%	63%	58%

^i^ The % represent the overall efficiency of the particular level of theory. The method of their computation is detailed in the section “Materials and Methods”.

**Table 2:** Validation set for BDE values (in kcal/mol) at M06–2X/6–311G(d,p).

Substance	BDE_calc_	BDE_exp_ [22]	Δ(BDE_calc_ − BDE_exp_)
Catechin	76.9	83.2 (C4′)	−6.3
Chrysin	92.6	85.4 (C7)	7.2
(–)-Epicatechin	82.4	82.0 (C4′)	0.4
(–)-Epigallocatechin	79.4	82.4 (C4′)	−3.0
Fisetin	86.5	83.2 (C4′)	3.3
Galangin	92.5	86.8 (C7)	5.7
Gallic acid	81.0	83.0 (C4)	−2.0
Luteolin	78.1	81.9 (C4′)	−3.8
Myricetin	79.6	81.5 (C4′)	−1.9
Quercetin	78.6	82.0 (C4′)	−3.4
Taxifolin	86.6	82.1 (C4′)	4.5
**MAE: 3.8 kcal/mol**		**RMSE: 4.2 kcal/mol**	

**Table 3:** List of functionals tested within the paper.

Method	Type	%HF (SR/MR/LR)
BLYP [30,31]	GGA	(0%)/0%/(0%)
TPSSh [32,33]	GH meta–GGA	(10%)/10%/(10%)
B3LYP [31,34]	GH GGA	(20%)/20%/(20%)
PW6B95 [35]	GH meta–GGA	(28%)/28%/(28%)
MPWB1K [36]	GH meta–GGA	(44%)/44%/(44%)
M06–2X [21]	GH meta–GGA	(54%)/54%/(54%)
WB97 [37]	RSH GGA	0%/0%/100%
WB97X [37]	RSH GGA	15.77%/0%/100%
CAM–B3LYP [38]	RSH GGA	19%/0%/65%
M11 [39]	RSH meta–GGA	42.8%/0%/100%
HISSbPBE [40]	RSH GGA	0%/60%/0%

**Table 4:** List of basis sets tested within the paper.

Family	Basis Set	Number of Basis Functions
Pople’s [42,43]	6–31G(d,p)	235
	6–31+G(d,p) [41,44,45,46]	287
	6–31++G(d,p)	295
	6–311G(d,p)	282
	6–311+G(d,p) [47,48]	334
	6–311++G(d,p)	342
Dunning’s [49,50]	cc–pVDZ	222
	aug–cc–pVDZ	371
	cc–pVTZ	502
	aug–cc–pVTZ	782
Ahlrich’s [51,52]	def2–SVP	222
	def2–SVPD	336
	def2–TZVP	451
	def2–TZVPD	565

## Data Availability

Supplementary Materials associated with this paper.

## Supplementary Materials

The following are available online, Cartesian coordinates of each structure produced, geometrical measurements, thermochemical values, HOMO and LUMO orbital energies. Additionally, a PDF file regarding conformational analysis in levels of theory under consideration is provided: Figure S1:” Relative Errors to the Reference Value for C3 (upper) and C4 (lower) Hydroxyl Bond Length. [Å].”, Figure S2: “Relative Errors to the Reference Value for Calculated Hydrogen Bond Length. [Å].”.
