# Supplementary material for: A Statistically Supported Antioxidant Activity DFT Benchmark—The Effects of Hartree–Fock Exchange and Basis Set Selection on Accuracy and Resources Uptake
Source: Molecules. 2021 Aug 20;26(16):5058. doi: 10.3390/molecules26165058 (PMC8398206; doi:10.3390/molecules26165058)
Supplement: Supplementary file 1 [file molecules-26-05058-s001.zip › SupplementaryMaterials_Spiegel2021/Conformational Analysis Protocol.pdf]

Conformational Analysis Protocol for

A Statistically Supported Antioxidant Activity DFT Benchmark — The Effects of Hartree–Fock Exchange and Basis Set Selection on Accuracy and Resource Uptake

Maciej Spiegel[1,*], Andrzej Gamian[2] and Zbigniew Sroka[1]

[1]*Department of Pharmacognosy and Herbal Medicines, Wroclaw Medical University, Borowska 211A, 50–556 Wroclaw, Poland*
[2]*Hirszfeld Institute of Immunology and Experimental Therapy, Polish Academy of Sciences, Rudolfa Weigla 12, 53–114 Wroclaw, Poland.*

* maciej.spiegel@student.umed.wroc.pl

In this supplementary material, we focused solely on the geometrical features that are also relevant to the observed antioxidative/antiradical behaviour. These are undoubtedly the O–H bond lengths of hydroxyl residues, the hydrogen bond lengths between hydroxyl groups, and the dihedral angles. A shorter bond length, in general, means that more energy is contained within it, and thus a higher value of energy is needed to break it. This may be expressed in polyphenols' proton or hydrogen detachment reaction enthalpies, and hence in the viability of reaction channels described by them.

Furthermore, the antiradical activity is maintained to a certain extent by the stabilization of the radical created. The formation of intramolecular and intermolecular hydrogen bonds, as well as the delocalization of the unpaired electron, accomplishes this. According to the studies, the first property is essential in the stabilization of the radical created. [1,2] This effect is amplified where three neighbouring hydroxyl groups are involved, with the middle one being the preferred abstraction site due to the two hydrogen bonds formed with the radical group. Following that, the values of the $O_{hydroxyl}\cdots(H–O)_{hydroxyl}$ hydrogen bond were determined.

Finally, the degree of p_y conjugation tends to be proportional to the lower energy of the radical forming reaction, as previously mentioned. This is not always so — in certain flavonoids, such as morin, the adjacency of the two hydroxyl groups at C3 and C2' produces a gradient that draws away from the planarity. However, the energy appears to be compensated by the hydrogen bond formed. [3] Nonetheless, since only the *p* shell is involved in the delocalization process for organic molecules composed of atoms from rows 1–3 of the periodic table, bond conjugation can be handled satisfactorily in a simple geometrical fashion. Bending from planarity occurs at three nodes in the case of caffeic acid: two bonds linking the aromatic ring to the hydroxyl residue and one bond connecting the aromatic ring to the side chain. That is why we investigated the torsion angles of $(C–C)_{ring}–(O–H)_{hydroxyl}$ and $(C–C)_{ring}–(C–C)_{side}$.

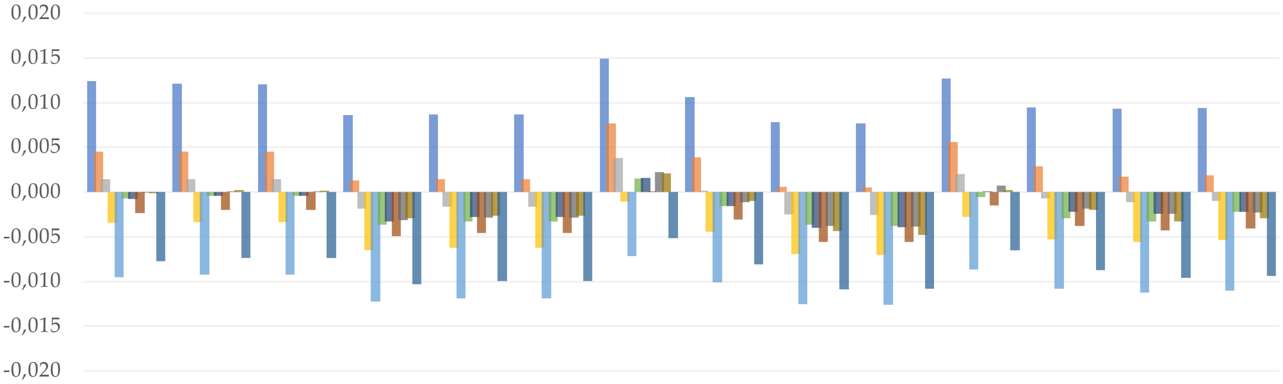

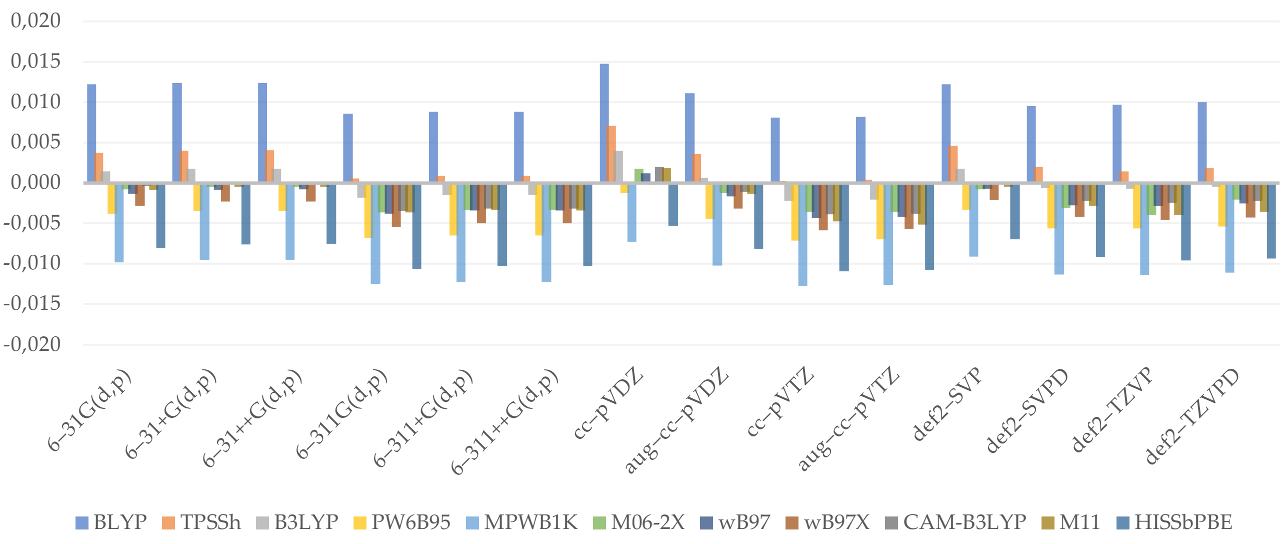

Figure S1. Relative Errors to the Reference Value for C3 (upper) and C4 (lower) Hydroxyl Bond Length. [Å].

Hydroxyl Bonds

According to the computational findings (**Figure S1**), most functionals tend to underestimate the bond length in hydroxyl groups. BLYP (*C3: 0.010Å; C4: 0.105Å*) and MPWB1K (*C3: 0.011Å; C4: 0.011Å*) had the highest MAE values, whereas B3LYP (*C3: 0.002Å; C4: 0.002Å*) and CAM–B3LYP (*C3: 0.002Å; C4: 0.002Å*) provided the lowest one.

Looking at the graph, one can see that bond lengths among GHs appear to shorten as the %HF increases, and begin to be underestimated above about 20%HF, a B3LYP-designated threshold. Surprisingly, considering 54%HF of Thrular's M06–2X, it produced a relatively decent outcome (*C3: 0.002Å; C4: 0.002Å*). This is not the only instance in which this functional distinguishes itself, as will be shown later.

Let us now look at the significance of the separation scheme by considering the RSHs. Most of them produce results that are comparable to those obtained for B3LYP and PW6B95, making them a very viable alternative, especially the aforementioned CAM–B3LYP. HISSbPBE is the only exception, since it significantly underestimates bond length; it is also the only functional involving middle–range interactions and not including short– and long–range exchange–correlation terms. However, before examining the impact of the former, we will first take a look at the short and long ones. When we examine the effect of %HF at short–range, for example, by comparing WB97 with WB97X, the disparity is not substantial but visible, manifesting itself in a shorter bond length. However, involving the M11 functional, which got a nearly three times greater value of %HF for the short–range term, the results tend to mimic those obtained for the WB97. Since the only distinction between them is a Laplacian in Minnesota functional, we believe this is one, if not the primary, explanation for the observed result.

Nonetheless, to investigate the effect of variations in the long–range term, WB97X was contrasted with CAM–B3LYP, since they all belong to GGA and, due to the comparatively minor influence of the short term shown earlier, the small disparity herein can be ignored. A 35% decrease in long–range within CAM–B3LYP was discovered to increase bond length, which leads to greater overall precision of that functional.

Results collected at HISSbPBE can be presented in a related fashion. Despite the fact that it is only composed of the middle–range term, it produces values that are significantly lower than those yielded by CAM–B3LYP. Since a reduction in the long term was found to trigger bond elongation, the observable behaviour shows that the middle–range term has a much stronger effect on the final outcome. The proposed investigation led us to the conclusion that the increasing %HF value at short–range have the lowest, negative influence; at long–range it has a larger and positive impact; and at the middle–range it had a substantial negative effect.

Moving on to basis sets, we can see two things: the first is a clear decrease in expected bond length as we move from double– to triple–ζ, which is particularly noticeable in Dunning's family. The second is that diffuse function has little effect unless cc–pVDZ, def2–SVP, and their augmented versions are considered. In reality, the last basis set has the lowest MAE (*C3: 0.004Å; C4: 0.004Å*), as do 6–31+G** and 6–31++G** (both *C3: 0.004Å; C4: 0.004Å*).

$$Y_{C3-OH} = 4.846 \times 10^{-5}\boldsymbol{SR}^{**} - 2.279 \times 10^{-4}\boldsymbol{MR}^{***} - 9.567 \times 10^{-5}\boldsymbol{LR}^{***} + 2.969 \times 10^{-3}\boldsymbol{\zeta}^{***} + 4.539 \times 10^{-3^{***}} \quad \text{(Eq. S1)}$$

$$Y_{C4-OH} = 5.034 \times 10^{-5}\boldsymbol{SR}^{**} - 2.281 \times 10^{-4}\boldsymbol{MR}^{***} - 9.962 \times 10^{-5}\boldsymbol{LR}^{***} + 2.871 \times 10^{-3}\boldsymbol{\zeta}^{***} + 4.538 \times 10^{-3^{***}} \quad \text{(Eq. S2)}$$

The linear regression models (**Equation S1** and **Equation S2**) were built and found to have R^2 values of 0.6631 and 0.6546, respectively, residual standard deviations of 0.003 in both cases, and p–values for *F*–statistics less than 0.05, suggesting statistical significance. According to them, the best predictors of bond lengths in the hydroxyl group are %HF values at each range and type of basis collection. The position of the middle-range term, which was suggested during the debate, is confirmed by the models' coefficients, as is the decrease in bond length with shift to triple–ζ. Surprisingly, despite the fact that the long–range term has a larger effect than the short–range term, the models forecast their signs of their coefficients oppositely of our assumptions. Short–range terms appear to be responsible for the bond length increase, whereas middle– and long–range terms appear to be responsible for decreasing bond length, with a focus placed on the middle range, which is one point greater than the other two, showing how a relatively minor shift in the %HF and separation scheme may have a major effect on the feature examined. The model perfectly explains how bond length decreases by moving from double– to triple–ζ. Aside from the intercept, the only one that is influenced purely by a %HF at medium–range and basis set type is the functional HISSbPBE. For C3, the combination HISSbPBE/6–31G(d,p) yields a relative error of –0.008Å, while HISSbPBE/6–311G(d,p) produces a relative error of –0.010Å. Thus, the difference is –0.003Å, which is almost equal to the coefficient value for triple– ζ. The presented scenario also occurs for the C4 model.

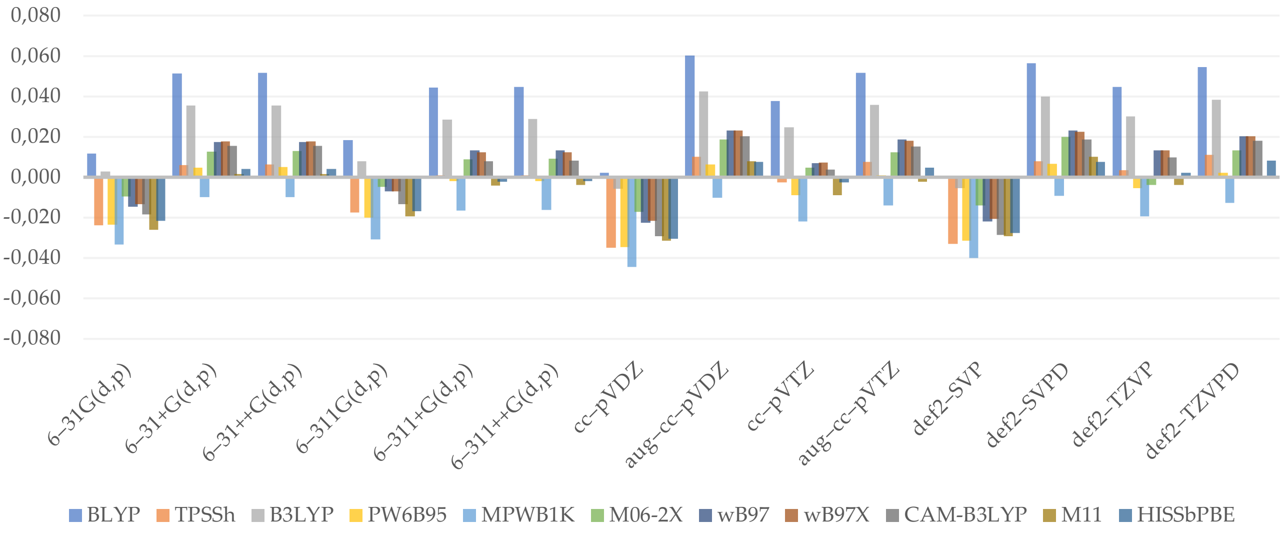

Figure S2. Relative Errors to the Reference Value for Calculated Hydrogen Bond Length. [Å].

Hydrogen Bonds

In the case of hydrogen bond length computations (**Figure S2**), no clear pattern can be observed, as was possible in the previous case. BLYP recorded the worse results (*0.038Å*) in the practical trials, while PW6B95 (*0.011Å*), as well as two RSHs — M11 (*0.017Å*) and HISSbPBE (*0.010Å*) — produced the highest. Surprisingly, the often-used B3LYP functional finished second to last in terms of accuracy

(*0.026Å*). No conclusions about the effect of %HF on hydrogen bond length can be reached explicitly from the values obtained. For example, how can we understand why TPSSh yields lower results than BLYP or B3LYP? There is no order in which BLYP and B3LYP differ from TPPSh, PW6B95, and MPWB1K. Disagreements can arise as a result of variations in how and which terms, in a scope of separation scheme, have the greatest influence on the final outcome.

At first glance, the visual representation of the findings shows that, in comparison to GHs, RSHs tend to produce values at the same degree. Functionals that differ in the short term, respectively WB97 and WB97X, appear to yield virtually equal results, with the second generating a slightly better one. That suggests that the short–range term has only a slight impact. Based on this premise, we can conclude that CAM–B3LYP and WB97X differ only in the long–range. As a result, decreasing the long–range term causes a minor, but noticeable, change in the value, resulting in a reduction in the length of the hydrogen bond. When HISSbPBE is investigated as a representative functional of the medium–range term, it usually produces very good results, to the same extent as M11. Unless paired with double–ζ, their results are more accurate than either of the previously mentioned functionals. In the case of CAM–B3LYP, this will imply that the medium range is responsible for the decrease in hydrogen bond length.

From there, it appears prudent to begin investigation how basis set choice influences the result, since it could play a larger role than functionals in the case of the property under consideration. In general, the plots are more systematized in their case — the inclusion of the diffuse function, as well as the transition from double– to triple–ζ, is easily apparent to improve the outcomes. The latter seems to have a smaller effect in the case of Pople's basis set but a greater effect on the results obtained by Dunning's and Ahlrich's. Moreover, cc–pVTZ and def2–TZVP tend to perform significantly better than diffused versions of any of the base sets in their family. It should also be noted that in the case of 6–31G or 6–311G basis sets, there is little distinction between single– or double–diffused versions, so a general suggestion of using the first is made in light of the computational resources' usage. Overall, the approximate MAE values point to two Dunning's functionals as extrema: minimum cc–pVTZ (*0.012Å*) and maximum cc–pVDZ (*0.025Å*).

$$\begin{aligned} \boldsymbol{Y_{OH\cdots OH}} = -1.653\times10^{-4}\boldsymbol{SR}^{*} - 3.421\times10^{-4}\boldsymbol{MR}^{***} - 1.666\times10^{-4}\boldsymbol{LR}^{***} \\ + 2.642\times10^{-5}\boldsymbol{\zeta}^{***} + 2.127\times10^{-2}\boldsymbol{D}^{***} \end{aligned} \quad (6)$$

The measurable influence of the diffusion is numerically clear from the proven regression equation (**Equation 6**). The coefficient responsible for its appearance is the largest, distinguishing from the others by at least two orders of magnitude. Interestingly, the model points that shift from double– to triple– ζ are negligible — which is in stark contrast to the figure's dependencies. In any case, the statistical definition of the separation schemes indicates that they are inversely proportional to the length of the hydrogen bond. While its short–term importance is smaller than the others, its removal resulted in a lower R^2 value. The factor for the medium–range term is almost twice as large as that estimated for the short– and long–range terms. According to the model's coefficients, the outcomes of GHs should steadily decrease as the %HF increases, but this does not happen. Furthermore, it agrees with the debate on HISSbPBE and the lowering effect of the medium–range term, but on the other hand, we ought to see that first, the exponent is so low that it should not be so apparent, and second, the discrepancies between WB97X and WB97 or CAM–B3LYP should be visible. These anomalies may be attributed to the very low R^2 value, which is lower than in the case of hydroxyl bond length, and equals 0.5482. These may be the result of a much more complicated statistical description of the observed hydrogen bond lengths. Aside from that, residual standard error was found to be minimal, 0.01488, and *F*–statistics meets the criterion of $p<0.05$, maintaining statistical significance of the model.

Conclusions

Despite our stringent comparative criteria, the overall results are generally acceptable, and the structures produced do not deviate significantly from the reference structure. Similar observations were made by the scientists benchmarking another common polyphenol – gallic acid.[4] We would also

like to note that all combinations correctly determined dihedral angles and hence can be claimed to satisfyingly reflect delocalization, at least geometrically.

The statistically significant effect on geometry is provided by the medium– and long–range terms of the functionals and diffusion on the basis set, according to the models developed. The role of the intercept was discovered to be important in the case of hydroxyl bonds' model, suggesting that some crucial features were ignored in our model. Despite this, our findings can be seen as a starting point for future research on more complex polyphenols, such as tannins. Tiny variations discovered in our caffeic acid case may emerge there intensely, drastically altering the geometry of the final structure, which is especially true where a large number of flexible bonds and residues are present.

---

1. Chen, Y.; Xiao, H.; Zheng, J.; Liang, G. Structure-thermodynamics-antioxidant activity relationships of selected natural phenolic acids and derivatives: An experimental and theoretical evaluation. *PLoS One* **2015**, *10*, 1–20.
2. Jeremić, S.; Radenković, S.; Filipović, M.; Antić, M.; Amić, A.; Marković, Z. Importance of hydrogen bonding and aromaticity indices in QSAR modeling of the antioxidative capacity of selected (poly)phenolic antioxidants. *J. Mol. Graph. Model.* **2017**, *72*, 240–245.
3. Spiegel, M.; Andruniów, T.; Sroka, Z. Flavones' and flavonols' antiradical structure– activity relationship—a quantum chemical study. *Antioxidants* **2020**, *9*, 461.
4. De Souza, G.L.C.; Peterson, K.A. Benchmarking Antioxidant-Related Properties for Gallic Acid through the Use of DFT, MP2, CCSD, and CCSD(T) Approaches. *J. Phys. Chem. A* **2021**, *125*, 198–208.
