# Supplementary material for: A Statistically Supported Antioxidant Activity DFT Benchmark—The Effects of Hartree–Fock Exchange and Basis Set Selection on Accuracy and Resources Uptake
Source: Molecules. 2021 Aug 20;26(16):5058. doi: 10.3390/molecules26165058 (PMC8398206; doi:10.3390/molecules26165058)
Supplement: Supplementary file 1 [file molecules-26-05058-s001.zip › SupplementaryMaterials_Spiegel2021/SupplementaryMaterials.pdf]

Supplementary Materials

for

A Statistically Supported Antioxidant Activity DFT Benchmark — The Effects of Hartree–Fock Exchange and Basis Set Selection on Accuracy and Resource Uptake

Maciej Spiegel[1,*], Andrzej Gamian[2] and Zbigniew Sroka[1]

[1]*Department of Pharmacognosy and Herbal Medicines, Wroclaw Medical University, Borowska 211A, 50–556 Wroclaw, Poland*
[2]*Hirszfeld Institute of Immunology and Experimental Therapy, Polish Academy of Sciences, Rudolfa Weigla 12, 53–114 Wroclaw, Poland.*

* maciej.spiegel@student.umed.wroc.pl

1. THEORETICAL REFERENCE DATA: 2
2. GEOMETRICAL MEASUREMENTS 3
  2.1. *C3–O–H Bond Length [Å]* 3
  2.2. *C4–O–H Bond Length [Å]* 4
  2.3. *O–H···O–H Bond Length [Å]* 5
  2.4. *(C2–C3)–(O–H) Dihedral Angle [°]* 6
  2.5. *(C3–C4)–(O–H) Dihedral Angle [°]* 7
  2.6. *(C2–C1)–(C'=C') Dihedral Angle [°]* 8
3. THERMOCHEMISTRY 9
  3.1. *Ground State Enthalpies [au]* 9
  3.2. *Relaxed C3 Radical Enthalpies [au]* 10
  3.3. *Relaxed C4 Radical Enthalpies [au]* 11
  3.4. *Relaxed Cationradical Enthalpies [au]* 12
  3.5. *Relaxed Anionradical Enthalpies [au]* 13
  3.6. *Relaxed C3 Anion Enthalpies [au]* 14
  3.7. *Relaxed C4 Anion Enthalpies [au]* 15
4. ORBITAL ENERGIES 16
  4.1. *HOMO [eV]* 16
  4.2. *LUMO [eV]* 17

1. Theoretical Reference Data:

Property	Value
(C3)O–H Bond Length [Å]	0,96761
(C4)O–H Bond Length [Å]	0,96404
O–H···O–H Bond Length [Å]	2,1235
(C2–C3)–(O–H) Dihedral Angle [°]	179,96989
(C3–C4)–(O–H) Dihedral Angle [°]	179,85515
(C2–C1)–(C=C) Dihedral Angle [°]	0,18752
Ground State Enthalpy [au]	-648,363763
Relaxed C3 Radical Enthalpy [au]	-647,718584
Relaxed C4 Radical Enthalpy [au]	-647,738512
Relaxed Cationradical Enthalpy [au]	-648,071505
Relaxed Anionradical Enthalpy [au]	-648,383006
Relaxed C3 Anion [au]	-647,823828
Relaxed C4 Anion [au]	-647,855579
HOMO [eV]	-7.1638
LUMO [eV]	-0.6659

2. Geometrical Measurements

2.1. C3–O–H Bond Length [Å]

	BLYP	TPSSh	B3LYP	PW6B95	MPWB1K	M06-2X	wB97	wB97X	CAM-B3LYP	M11	HISSbPBE
**6–31G(d,p)**	0,98001	0,97212	0,96903	0,96416	0,95806	0,96688	0,9668	0,96525	0,96757	0,96746	0,95988
**6–31+G(d,p)**	0,97972	0,97214	0,96906	0,96428	0,95834	0,96719	0,96722	0,96561	0,96768	0,96780	0,96023
**6–31++G(d,p)**	0,97969	0,97214	0,96904	0,96427	0,95834	0,96718	0,96721	0,96560	0,96767	0,96778	0,96023
**6–311G(d,p)**	0,97624	0,96889	0,96576	0,96111	0,95538	0,96399	0,96434	0,96265	0,96447	0,96466	0,95730
**6–311+G(d,p)**	0,97632	0,96907	0,96598	0,96138	0,95569	0,96429	0,96479	0,96303	0,96474	0,96498	0,95763
**6–311++G(d,p)**	0,97632	0,96908	0,96599	0,96138	0,95569	0,96430	0,96479	0,96303	0,96475	0,96498	0,95763
**cc–pVDZ**	0,98254	0,97532	0,97143	0,96654	0,96048	0,96915	0,96918	0,96765	0,96987	0,96972	0,96246
**aug–cc–pVDZ**	0,97820	0,97149	0,96778	0,96316	0,95748	0,96604	0,96607	0,96452	0,96647	0,96660	0,95948
**cc–pVTZ**	0,97542	0,96820	0,96507	0,96063	0,95504	0,96395	0,96363	0,96199	0,96381	0,96325	0,95672
**aug–cc–pVTZ**	0,97529	0,96812	0,96502	0,96056	0,95502	0,96385	0,96368	0,96201	0,96377	0,96284	0,95678
**def2–SVP**	0,98033	0,97321	0,96962	0,96482	0,95897	0,96703	0,96766	0,96614	0,96830	0,96782	0,96110
**def2–SVPD**	0,97707	0,97048	0,96690	0,96233	0,95679	0,96465	0,96541	0,96383	0,96574	0,96558	0,95888
**def2–TZVP**	0,97694	0,96932	0,96649	0,96205	0,95638	0,96435	0,96519	0,96328	0,96516	0,96435	0,95801
**def2–TZVPD**	0,97704	0,96951	0,96663	0,96221	0,95655	0,96539	0,96541	0,96351	0,96532	0,96469	0,95823

2.2. C4–O–H Bond Length [Å]

	6–31G(d,p)	6–31+G(d,p)	6–31++G(d,p)	6–311G(d,p)	6–311+G(d,p)	6–311++G(d,p)	cc–pVDZ	aug–cc–pVDZ	cc–pVTZ	aug–cc–pVTZ	def2–SVP	def2–SVPD	def2–TZVP	def2–TZVPD
**BLYP**	0,9763	0,97640	0,97643	0,97258	0,97288	0,97288	0,97881	0,97515	0,97213	0,97224	0,97623	0,97357	0,97374	0,97402
**TPSSh**	0,96775	0,96805	0,96808	0,96463	0,96493	0,96493	0,97112	0,96766	0,96428	0,96445	0,96865	0,96606	0,96549	0,96584
**B3LYP**	0,96552	0,96578	0,9658	0,96227	0,96258	0,96258	0,96800	0,96471	0,96186	0,96198	0,96583	0,96342	0,96333	0,96360
**PW6B95**	0,96029	0,96058	0,96060	0,95726	0,95757	0,95757	0,96279	0,95964	0,95696	0,95709	0,96074	0,95839	0,95840	0,95868
**MPWB1K**	0,95419	0,95456	0,95458	0,95151	0,95181	0,95181	0,95677	0,95384	0,95131	0,95145	0,95495	0,95274	0,95265	0,95293
**M06-2X**	0,96324	0,96357	0,96359	0,96042	0,96069	0,96069	0,96581	0,96279	0,96049	0,96049	0,96328	0,96099	0,96008	0,96199
**wB97**	0,96267	0,96322	0,96323	0,96025	0,96068	0,96068	0,96526	0,96237	0,95971	0,95989	0,96337	0,96131	0,96121	0,96155
**wB97X**	0,96121	0,96172	0,96174	0,95862	0,95903	0,95903	0,96379	0,96092	0,95820	0,95835	0,96191	0,95982	0,95943	0,95979
**CAM-B3LYP**	0,96365	0,96398	0,96400	0,96055	0,96088	0,96088	0,96602	0,96294	0,96014	0,96028	0,96408	0,96181	0,96156	0,96183
**M11**	0,96316	0,96358	0,96360	0,96040	0,96069	0,96068	0,96591	0,96274	0,95928	0,95889	0,96359	0,96121	0,96008	0,96047
**HISSbPBE**	0,956	0,95648	0,95650	0,95343	0,95377	0,95377	0,95877	0,95588	0,95309	0,95329	0,95707	0,95489	0,95443	0,95474

2.3. O–H···O–H Bond Length [Å]

	6–31G(d,p)	6–31+G(d,p)	6–31++G(d,p)	6–311G(d,p)	6–311+G(d,p)	6–311++G(d,p)	cc–pVDZ	aug–cc–pVDZ	cc–pVTZ	aug–cc–pVTZ	def2–SVP	def2–SVPD	def2–TZVP	def2–TZVPD
**BLYP**	2,13508	2,17497	2,17527	2,14173	2,16801	2,16831	2,12570	2,18361	2,16106	2,17529	2,12399	2,17993	2,16802	2,17798
**TPSSh**	2,09982	2,12959	2,12982	2,10589	2,12429	2,12443	2,08866	2,13368	2,12089	2,13113	2,09064	2,13144	2,12681	2,13462
**B3LYP**	2,1264	2,15885	2,15906	2,13136	2,15207	2,15225	2,11785	2,16580	2,14833	2,15936	2,11799	2,16352	2,15368	2,16181
**PW6B95**	2,09994	2,12827	2,12843	2,10348	2,12142	2,12165	2,08898	2,12974	2,11450	2,12438	2,09213	2,13006	2,11810	2,12576
**MPWB1K**	2,09004	2,11351	2,11362	2,09263	2,10702	2,10724	2,07899	2,11333	2,10166	2,10940	2,08356	2,11444	2,10412	2,11065
**M06-2X**	2,11385	2,13624	2,13632	2,11866	2,13247	2,13263	2,10650	2,14221	2,12820	2,13589	2,10967	2,14334	2,11959	2,13668
**wB97**	2,10876	2,14093	2,14095	2,11648	2,13689	2,13692	2,10087	2,14668	2,13053	2,14218	2,10176	2,14652	2,13667	2,14370
**wB97X**	2,1103	2,14110	2,14116	2,11649	2,13578	2,13583	2,10200	2,14660	2,13062	2,14149	2,10288	2,14601	2,13692	2,14379
**CAM-B3LYP**	2,10495	2,13887	2,13901	2,11029	2,13154	2,13179	2,09439	2,14384	2,12727	2,13866	2,09494	2,14230	2,13346	2,14166
**M11**	2,09734	2,12499	2,12508	2,10403	2,11947	2,11975	2,09194	2,13153	2,11464	2,12139	2,09443	2,13370	2,11959	2,12367
**HISSbPBE**	2,10198	2,12761	2,12775	2,10664	2,12118	2,12151	2,09294	2,13119	2,12090	2,12836	2,09578	2,13110	2,12556	2,13161

2.4. (C2–C3)–(O–H) Dihedral Angle [°]

	6–31G(d,p)	6–31+G(d,p)	6–31++G(d,p)	6–311G(d,p)	6–311+G(d,p)	6–311++G(d,p)	cc–pVDZ	aug–cc–pVDZ	cc–pVTZ	aug–cc–pVTZ	def2–SVP	def2–SVPD	def2–TZVP	def2–TZVPD
**BLYP**	0,0	0,0	0,0	0,0	0,0	0,0	0,0	0,0	0,0	0,0	0,0	0,0	0,0	0,0
**TPSSh**	0,0	0,0	0,0	0,0	0,0	0,0	0,0	0,0	0,0	0,0	0,0	0,0	0,0	0,0
**B3LYP**	0,0	0,0	0,0	0,0	0,0	0,0	0,0	0,0	0,0	0,0	0,0	0,0	0,0	0,0
**PW6B95**	0,0	0,0	0,0	0,0	0,0	0,0	0,0	0,0	0,0	0,0	0,0	0,0	0,0	0,0
**MPWB1K**	0,0	0,0	0,0	0,0	0,0	0,0	0,0	0,0	0,0	0,0	0,0	0,0	0,0	0,0
**M06-2X**	0,0	0,0	0,0	0,0	0,0	0,0	0,0	0,0	0,0	0,0	0,0	0,0	0,0	0,0
**wB97**	0,0	0,0	0,0	0,0	0,0	0,0	0,0	0,0	0,0	0,0	0,0	0,0	0,0	0,0
**wB97X**	0,0	0,0	0,0	0,0	0,0	0,0	0,0	0,0	0,0	0,0	0,0	0,0	0,0	0,0
**CAM-B3LYP**	0,0	0,0	0,0	0,0	0,0	0,0	0,0	0,0	0,0	0,0	0,0	0,0	0,0	0,0
**M11**	0,0	0,0	0,0	0,0	0,0	179,4	0,0	0,0	0,0	0,0	0,0	0,0	0,0	0,0
**HISSbPBE**	0,0	0,0	0,0	0,0	0,0	0,0	0,0	0,0	0,0	0,0	0,0	0,0	0,0	0,0

2.5. (C3–C4)–(O–H) Dihedral Angle [°]

	BLYP	TPSSh	B3LYP	PW6B95	MPWB1K	M06-2X	wB97	wB97X	CAM-B3LYP	M11	HISSbPBE
**6–31G(d,p)**	0,0	0,0	0,0	0,0	0,0	0,0	0,0	0,0	0,0	0,0	0,0
**6–31+G(d,p)**	0,0	0,0	0,0	0,0	0,0	179,9	179,9	0,0	0,0	0,0	0,0
**6–31++G(d,p)**	0,0	0,0	0,0	0,0	0,0	0,0	0,0	0,0	0,0	0,0	0,0
**6–311G(d,p)**	0,0	0,0	0,0	0,0	0,0	0,0	0,0	0,0	0,0	0,0	0,0
**6–311+G(d,p)**	0,0	0,0	0,0	0,0	0,0	0,0	0,0	0,0	0,0	0,0	0,0
**6–311++G(d,p)**	0,0	0,0	0,0	0,0	0,0	0,0	0,0	0,0	0,0	179,8	0,0
**cc–pVDZ**	0,0	0,0	0,0	0,0	0,0	0,0	0,0	0,0	0,0	0,0	0,0
**aug–cc–pVDZ**	0,0	0,0	0,0	0,0	0,0	0,0	0,0	0,0	0,0	0,0	0,0
**cc–pVTZ**	0,0	0,0	0,0	0,0	0,0	0,0	0,0	0,0	0,0	0,0	0,0
**aug–cc–pVTZ**	0,0	0,0	0,0	0,0	0,0	0,0	0,0	0,0	0,0	0,0	0,0
**def2–SVP**	0,0	0,0	0,0	0,0	0,0	0,0	0,0	0,0	0,0	0,0	0,0
**def2–SVPD**	0,0	0,0	0,0	0,0	0,0	0,0	0,0	0,0	0,0	0,0	0,0
**def2–TZVP**	0,0	0,0	0,0	0,0	0,0	0,0	0,0	0,0	0,0	0,0	0,0
**def2–TZVPD**	0,0	0,0	0,0	0,0	0,0	0,0	0,0	0,0	0,0	0,0	0,0

2.6. (C2–C1)–(C'=C') Dihedral Angle [°]

HISSbPBE	M11	CAM-B3LYP	wB97X	wB97	M06-2X	MPWB1K	PW6B95	B3LYP	TPSSh	BLYP	
0,0	0,0	0,0	0,0	0,0	0,0	0,0	0,0	0,0	0,0	0,0	**6–31G(d,p)**
0	0,0	0,0	0,0	1,1	2,8	0,0	0,0	0,0	0,0	0,0	**6–31+G(d,p)**
0,0	0,0	0,0	0,0	0,0	0,0	0,0	0,0	0,0	0,0	0,0	**6–31++G(d,p)**
0,0	0,0	0,0	0,0	0,0	0,0	0,0	0,0	0,0	0,0	0,0	**6–311G(d,p)**
-0,00149	0,0	0,0	0,0	0,0	0,0	0,0	0,0	0,0	0,0	0,0	**6–311+G(d,p)**
0	5,7	0,0	0,0	0,0	0,0	0,0	0,0	0,0	0,0	0,0	**6–311++G(d,p)**
0,00027	0,0	0,0	0,0	0,0	0,0	0,0	0,0	0,0	0,0	0,0	**cc–pVDZ**
0	0,0	0,0	0,0	0,0	0,0	0,0	0,0	0,0	0,0	0,0	**aug–cc–pVDZ**
0,0	0,0	0,0	0,0	0,0	0,0	0,0	0,00032	0,0	0,0	0,0	**cc–pVTZ**
0,0	0,0	0,0	0,0	0,0	0,0	0,0	0,0	0,0	0,0	0,0	**aug–cc–pVTZ**
0,0	0,0	0,0	0,0	0,0	0,0	0,0	0,0	0,0	0,0	0,0	**def2–SVP**
0	0,0	0,0	0,0	0,0	0,0	0,0	0,0	0,0	0,0	0,0	**def2–SVPD**
0,0	0,0	0,0	0,0	0,0	0,0	0,0	0,0	0,0	0,0	0,0	**def2–TZVP**
180	0,0	0,0	0,0	0,0	0,0	0,0	0,0	0,0	0,0	0,0	**def2–TZVPD**

3. Thermochemistry

3.1. Ground State Enthalpies [au]

	6–31G(d,p)	6–31+G(d,p)	6–31++G(d,p)	6–311G(d,p)	6–311+G(d,p)	6–311++G(d,p)	cc–pVDZ	aug–cc–pVDZ	cc–pVTZ	aug–cc–pVTZ	def2–SVP	def2–SVPD	def2–TZVP	def2–TZVPD
**BLYP**	-648,32694	-648,369	-648,369	-648,507	-648,529	-648,529	-648,360	-648,424	-648,575	-648,588	-647,848	-647,906	-648,596	-648,602
**TPSSh**	-648,53293	-648,562	-648,562	-648,694	-648,710	-648,711	-648,581	-648,628	-648,761	-648,771	-648,058	-648,101	-648,782	-648,787
**B3LYP**	-648,51283	-648,548	-648,548	-648,680	-648,699	-648,699	-648,547	-648,602	-648,750	-648,761	-648,031	-648,082	-648,770	-648,775
**PW6B95**	-649,30285	-649,334	-649,334	-649,464	-649,482	-649,482	-649,346	-649,397	-649,530	-649,541	-648,817	-648,863	-649,554	-649,558
**MPWB1K**	-648,21458	-648,240	-648,240	-648,366	-648,381	-648,382	-648,259	-648,304	-648,434	-648,443	-647,726	-647,767	-648,456	-648,460
**M06-2X**	-648,25008	-648,277	-648,277	-648,423	-648,438	-648,439	-648,317	-648,365	-648,499	-648,510	-647,765	-647,808	-648,471	-648,513
**wB97**	-648,39829	-648,429	-648,429	-648,558	-648,575	-648,575	-648,441	-648,491	-648,616	-648,629	-647,913	-647,960	-648,640	-648,645
**wB97X**	-648,34788	-648,377	-648,378	-648,504	-648,521	-648,521	-648,386	-648,434	-648,567	-648,578	-647,864	-647,909	-648,590	-648,594
**CAM-B3LYP**	-648,21643	-648,252	-648,252	-648,386	-648,405	-648,405	-648,251	-648,307	-648,458	-648,469	-647,737	-647,788	-648,479	-648,483
**M11**	-648,18883	-648,218	-648,219	-648,366	-648,382	-648,382	-648,227	-648,279	-648,462	-648,478	-647,684	-647,731	-648,471	-648,478
**HISSbPBE**	-647,89105	-647,917	-647,917	-648,045	-648,059	-648,060	-647,925	-647,973	-648,116	-648,126	-647,417	-647,461	-648,137	-648,141

3.2. Relaxed C3 Radical Enthalpies [au]

	6–31G(d,p)	6–31+G(d,p)	6–31++G(d,p)	6–311G(d,p)	6–311+G(d,p)	6–311++G(d,p)	cc–pVDZ	aug–cc–pVDZ	cc–pVTZ	aug–cc–pVTZ	def2–SVP	def2–SVPD	def2–TZVP	def2–TZVPD
**BLYP**	-647,70592	-647,746	-647,746	-647,884	-647,904	-647,904	-647,741	-647,800	-647,950	-647,963	-647,226	-647,281	-647,973	-647,978
**TPSSh**	-647,90331	-647,930	-647,931	-648,063	-648,078	-648,078	-647,953	-647,997	-648,129	-648,138	-647,427	-647,468	-648,151	-648,154
**B3LYP**	-647,88106	-647,914	-647,914	-648,047	-648,064	-648,064	-647,917	-647,969	-648,115	-648,126	-647,399	-647,447	-648,137	-648,141
**PW6B95**	-648,66899	-648,698	-648,698	-648,829	-648,845	-648,845	-648,714	-648,762	-648,893	-648,904	-648,182	-648,226	-648,918	-648,922
**MPWB1K**	-647,58129	-647,605	-647,605	-647,732	-647,746	-647,746	-647,627	-647,669	-647,798	-647,807	-647,091	-647,131	-647,821	-647,825
**M06-2X**	-647,61419	-647,640	-647,640	-647,785	-647,800	-647,800	-647,682	-647,727	-647,860	-647,870	-647,127	-647,169	-647,833	-647,875
**wB97**	-647,76648	-647,796	-647,797	-647,925	-647,942	-647,942	-647,811	-647,858	-647,982	-647,994	-647,281	-647,325	-648,008	-648,012
**wB97X**	-647,7148	-647,743	-647,744	-647,870	-647,886	-647,886	-647,754	-647,800	-647,932	-647,942	-647,230	-647,273	-647,956	-647,959
**CAM-B3LYP**	-647,58502	-647,619	-647,619	-647,753	-647,771	-647,771	-647,621	-647,674	-647,823	-647,834	-647,105	-647,154	-647,845	-647,849
**M11**	-647,55185	-647,579	-647,579	-647,727	-647,743	-647,743	-647,591	-647,640	-647,823	-647,839	-647,046	-647,092	-647,833	-647,840
**HISSbPBE**	-647,25527	-647,280	-647,280	-647,407	-647,421	-647,421	-647,291	-647,335	-647,477	-647,487	-646,781	-646,822	-647,499	-647,503

3.3. Relaxed C4 Radical Enthalpies [au]

	**6–31G(d,p)**	**6–31+G(d,p)**	**6–31++G(d,p)**	**6–311G(d,p)**	**6–311+G(d,p)**	**6–311++G(d,p)**	**cc–pVDZ**	**aug–cc–pVDZ**	**cc–pVTZ**	**aug–cc–pVTZ**	**def2–SVP**	**def2–SVPD**	**def2–TZVP**	**def2–TZVPD**
**BLYP**	-647,7269	-647,766	-647,766	-647,904	-647,924	-647,924	-647,762	-647,819	-647,970	-647,982	-647,247	-647,301	-647,992	-647,997
**TPSSh**	-647,92356	-647,950	-647,950	-648,082	-648,097	-648,098	-647,974	-648,017	-648,148	-648,157	-647,448	-647,488	-648,170	-648,174
**B3LYP**	-647,90054	-647,933	-647,933	-648,066	-648,083	-648,083	-647,937	-647,987	-648,134	-648,144	-647,419	-647,465	-648,155	-648,159
**PW6B95**	-648,68858	-648,717	-648,717	-648,848	-648,864	-648,864	-648,734	-648,781	-648,912	-648,923	-648,202	-648,245	-648,937	-648,941
**MPWB1K**	-647,6002	-647,624	-647,624	-647,750	-647,765	-647,765	-647,646	-647,688	-647,817	-647,826	-647,110	-647,150	-647,840	-647,843
**M06-2X**	-647,63161	-647,658	-647,657	-647,803	-647,817	-647,817	-647,700	-647,745	-647,878	-647,887	-647,146	-647,187	-647,851	-647,892
**wB97**	-647,78495	-647,813	-647,814	-647,943	-647,959	-647,959	-647,829	-647,876	-648,000	-648,012	-647,299	-647,343	-648,026	-648,029
**wB97X**	-647,73329	-647,760	-647,761	-647,888	-647,903	-647,903	-647,773	-647,818	-647,950	-647,960	-647,248	-647,291	-647,974	-647,977
**CAM-B3LYP**	-647,60412	-647,637	-647,637	-647,772	-647,789	-647,789	-647,640	-647,692	-647,842	-647,852	-647,124	-647,172	-647,864	-647,868
**M11**	-647,57009	-647,597	-647,598	-647,746	-647,761	-647,761	-647,610	-647,659	-647,841	-647,856	-647,065	-647,110	-647,851	-647,858
**HISSbPBE**	-647,27462	-647,299	-647,299	-647,426	-647,440	-647,440	-647,310	-647,354	-647,496	-647,506	-646,800	-646,841	-647,518	-647,522

3.4. Relaxed Cationradical Enthalpies [au]

	6–31G(d,p)	6–31+G(d,p)	6–31++G(d,p)	6–311G(d,p)	6–311+G(d,p)	6–311++G(d,p)	cc–pVDZ	aug–cc–pVDZ	cc–pVTZ	aug–cc–pVTZ	def2–SVP	def2–SVPD	def2–TZVP	def2–TZVPD
**BLYP**	-648,06576	-648,093	-648,093	-648,236	-648,250	-648,250	-648,096	-648,147	-648,302	-648,312	-647,582	-647,628	-648,322	-648,326
**TPSSh**	-648,26136	-648,280	-648,280	-648,416	-648,427	-648,427	-648,308	-648,347	-648,482	-648,490	-647,781	-647,819	-648,502	-648,505
**B3LYP**	-648,23814	-648,261	-648,261	-648,398	-648,410	-648,410	-648,270	-648,315	-648,466	-648,474	-647,752	-647,794	-648,485	-648,488
**PW6B95**	-649,02478	-649,045	-649,045	-649,180	-649,191	-649,192	-649,066	-649,109	-649,244	-649,253	-648,533	-648,573	-649,267	-649,270
**MPWB1K**	-647,93438	-647,951	-647,951	-648,081	-648,091	-648,092	-647,977	-648,016	-648,148	-648,155	-647,440	-647,478	-648,169	-648,172
**M06-2X**	-647,96342	-647,981	-647,981	-648,129	-648,140	-648,140	-648,029	-648,070	-648,204	-648,213	-647,473	-647,512	-648,176	-648,217
**wB97**	-648,11738	-648,137	-648,138	-648,271	-648,283	-648,283	-648,158	-648,200	-648,329	-648,339	-647,628	-647,668	-648,352	-648,355
**wB97X**	-648,06709	-648,086	-648,086	-648,217	-648,228	-648,228	-648,103	-648,144	-648,280	-648,288	-647,578	-647,617	-648,301	-648,304
**CAM-B3LYP**	-647,93782	-647,961	-647,961	-648,100	-648,113	-648,113	-647,970	-648,017	-648,170	-648,179	-647,453	-647,497	-648,189	-648,193
**M11**	-647,90036	-647,920	-647,920	-648,071	-648,082	-648,083	-647,938	-647,983	-648,168	-648,181	-647,393	-647,436	-648,176	-648,182
**HISSbPBE**	-647,61048	-647,628	-647,628	-647,758	-647,768	-647,769	-647,643	-647,684	-647,829	-647,837	-647,132	-647,171	-647,849	-647,852

3.5. Relaxed Anionradical Enthalpies [au]

	6–31G(d,p)	6–31+G(d,p)	6–31++G(d,p)	6–311G(d,p)	6–311+G(d,p)	6–311++G(d,p)	cc–pVDZ	aug–cc–pVDZ	cc–pVTZ	aug–cc–pVTZ	def2–SVP	def2–SVPD	def2–TZVP	def2–TZVPD
**BLYP**	-648,33083	-648,398	-648,400	-648,523	-648,558	-648,560	-648,369	-648,454	-648,595	-648,618	-647,859	-647,936	-648,621	-648,631
**TPSSh**	-648,54277	-648,590	-648,590	-648,713	-648,740	-648,740	-648,595	-648,658	-648,783	-648,800	-648,074	-648,130	-648,807	-648,814
**B3LYP**	-648,52216	-648,578	-648,578	-648,700	-648,731	-648,731	-648,561	-648,634	-648,773	-648,792	-648,047	-648,112	-648,797	-648,805
**PW6B95**	-649,31085	-649,361	-649,362	-649,481	-649,510	-649,510	-649,358	-649,426	-649,550	-649,569	-648,831	-648,890	-649,577	-649,585
**MPWB1K**	-648,22014	-648,263	-648,263	-648,379	-648,405	-648,405	-648,268	-648,327	-648,450	-648,466	-647,738	-647,791	-648,475	-648,482
**M06-2X**	-648,25894	-648,304	-648,304	-648,442	-648,467	-648,467	-648,330	-648,392	-648,522	-648,538	-647,780	-647,836	-648,498	-648,540
**wB97**	-648,40475	-648,455	-648,455	-648,573	-648,602	-648,602	-648,451	-648,518	-648,635	-648,654	-647,926	-647,986	-648,663	-648,670
**wB97X**	-648,35443	-648,403	-648,403	-648,520	-648,547	-648,548	-648,396	-648,461	-648,586	-648,603	-647,877	-647,935	-648,612	-648,619
**CAM-B3LYP**	-648,22411	-648,280	-648,280	-648,405	-648,435	-648,435	-648,262	-648,337	-648,479	-648,498	-647,751	-647,817	-648,503	-648,511
**M11**	-648,20082	-648,248	-648,249	-648,388	-648,413	-648,413	-648,242	-648,308	-648,486	-648,506	-647,700	-647,759	-648,498	-648,507
**HISSbPBE**	-647,90329	-647,946	-647,947	-648,065	-648,090	-648,090	-647,941	-648,003	-648,139	-648,155	-647,436	-647,491	-648,163	-648,169

3.6. Relaxed C3 Anion Enthalpies [au]

	HISSbPBE	M11	CAM-B3LYP	wB97X	wB97	M06-2X	MPWB1K	PW6B95	B3LYP	TPSSh	BLYP
**6–31G(d,p)**	-647,32789	-647,62836	-647,65368	-647,78322	-647,83355	-647,69018	-647,65142	-648,74228	-647,95382	-647,9759	-647,77383
**6–31+G(d,p)**	-647,370	-647,675	-647,710	-647,831	-647,883	-647,734	-647,693	-648,792	-648,009	-648,022	-647,839
**6–31++G(d,p)**	-647,371	-647,675	-647,710	-647,831	-647,883	-647,734	-647,694	-648,792	-648,009	-648,022	-647,839
**6–311G(d,p)**	-647,489	-647,816	-647,833	-647,948	-648,001	-647,872	-647,810	-648,912	-648,130	-648,145	-647,964
**6–311+G(d,p)**	-647,513	-647,840	-647,864	-647,975	-648,030	-647,896	-647,835	-648,940	-648,160	-648,171	-647,998
**6–311++G(d,p)**	-647,513	-647,840	-647,864	-647,975	-648,030	-647,897	-647,835	-648,940	-648,160	-648,171	-647,999
**cc–pVDZ**	-647,367	-647,671	-647,694	-647,827	-647,882	-647,763	-647,701	-648,791	-647,994	-648,029	-647,813
**aug–cc–pVDZ**	-647,427	-647,736	-647,766	-647,890	-647,947	-647,823	-647,758	-648,857	-648,064	-648,090	-647,894
**cc–pVTZ**	-647,563	-647,913	-647,908	-648,014	-648,063	-647,952	-647,880	-648,980	-648,203	-648,214	-648,035
**aug–cc–pVTZ**	-647,578	-647,933	-647,926	-648,031	-648,082	-647,967	-647,895	-648,998	-648,221	-648,230	-648,057
**def2–SVP**	-646,859	-647,126	-647,180	-647,304	-647,354	-647,210	-647,168	-648,262	-647,478	-647,506	-647,300
**def2–SVPD**	-646,914	-647,186	-647,247	-647,363	-647,415	-647,265	-647,220	-648,321	-647,543	-647,561	-647,375
**def2–TZVP**	-647,588	-647,927	-647,934	-648,041	-648,092	-647,927	-647,907	-649,009	-648,228	-648,239	-648,062
**def2–TZVPD**	-647,594	-647,935	-647,941	-648,048	-648,099	-647,970	-647,913	-649,016	-648,236	-648,246	-648,071

3.7. Relaxed C4 Anion Enthalpies [au]

	6–31G(d,p)	6–31+G(d,p)	6–31++G(d,p)	6–311G(d,p)	6–311+G(d,p)	6–311++G(d,p)	cc–pVDZ	aug–cc–pVDZ	cc–pVTZ	aug–cc–pVTZ	def2–SVP	def2–SVPD	def2–TZVP	def2–TZVPD
**BLYP**	-647,81031	-647,871	-647,872	-647,999	-648,031	-648,031	-647,849	-647,926	-648,069	-648,088	-647,337	-647,408	-648,095	-648,103
**TPSSh**	-648,0135	-648,056	-648,057	-648,181	-648,205	-648,205	-648,066	-648,123	-648,249	-648,264	-647,543	-647,595	-648,273	-648,280
**B3LYP**	-647,99035	-648,042	-648,042	-648,165	-648,193	-648,193	-648,030	-648,097	-648,237	-648,253	-647,514	-647,575	-648,261	-648,268
**PW6B95**	-648,77924	-648,826	-648,826	-648,947	-648,974	-648,974	-648,828	-648,890	-649,015	-649,031	-648,299	-648,354	-649,042	-649,049
**MPWB1K**	-647,68869	-647,728	-647,728	-647,846	-647,869	-647,869	-647,738	-647,792	-647,915	-647,929	-647,205	-647,255	-647,941	-647,946
**M06-2X**	-647,72529	-647,766	-647,767	-647,905	-647,928	-647,929	-647,797	-647,854	-647,984	-647,999	-647,245	-647,297	-647,959	-648,002
**wB97**	-647,86894	-647,916	-647,916	-648,035	-648,062	-648,062	-647,916	-647,978	-648,096	-648,113	-647,389	-647,447	-648,124	-648,130
**wB97X**	-647,81863	-647,863	-647,864	-647,982	-648,007	-648,007	-647,861	-647,921	-648,046	-648,062	-647,339	-647,395	-648,074	-648,079
**CAM-B3LYP**	-647,69055	-647,743	-647,744	-647,869	-647,897	-647,897	-647,730	-647,799	-647,942	-647,959	-647,216	-647,280	-647,967	-647,974
**M11**	-647,66436	-647,708	-647,709	-647,850	-647,872	-647,873	-647,706	-647,769	-647,946	-647,965	-647,162	-647,219	-647,959	-647,968
**HISSbPBE**	-647,36619	-647,406	-647,406	-647,526	-647,548	-647,548	-647,405	-647,461	-647,599	-647,612	-646,897	-646,949	-647,623	-647,628

4. Orbital Energies

4.1. HOMO [eV]

	6–31G(d,p)	6–31+G(d,p)	6–31++G(d,p)	6–311G(d,p)	6–311+G(d,p)	6–311++G(d,p)	cc–pVDZ	aug–cc–pVDZ	cc–pVTZ	aug–cc–pVTZ	def2–SVP	def2–SVPD	def2–TZVP	def2–TZVPD
**BLYP**	-0,17919	-0,19738	-0,19735	-0,17919	-0,19962	-0,19961	-0,18264	-0,19823	-0,19314	-0,19879	-0,18466	-0,19912	-0,19562	-0,19843
**TPSSh**	-0,20266	-0,21569	-0,21566	-0,21037	-0,21733	-0,2173	-0,20511	-0,21574	-0,2122	-0,21625	-0,20775	-0,21687	-0,21397	-0,21607
**B3LYP**	-0,2154	-0,2304	-0,23037	-0,22444	-0,23243	-0,23242	-0,21831	-0,23085	-0,22687	-0,2313	-0,22072	-0,23196	-0,22891	-0,23103
**PW6B95**	-0,22665	-0,24021	-0,24018	-0,23433	-0,24172	-0,24169	-0,22932	-0,24032	-0,23639	-0,24053	-0,23213	-0,24163	-0,23831	-0,24036
**MPWB1K**	-0,24903	-0,26017	-0,26014	-0,25537	-0,26146	-0,26142	-0,25127	-0,2599	-0,25676	-0,25975	-0,25451	-0,26144	-0,25835	-0,25884
**M06-2X**	-0,2637	-0,27519	-0,27516	-0,27195	-0,27776	-0,27774	-0,26568	-0,27467	-0,27453	-0,27749	-0,2684	-0,27596	-0,31623	-0,27643
**wB97**	-0,30593	-0,31916	-0,31913	-0,31306	-0,32059	-0,32058	-0,30801	-0,31899	-0,31518	-0,31886	-0,31082	-0,3205	-0,31727	-0,3187
**wB97X**	-0,29933	-0,31226	-0,31223	-0,3067	-0,31383	-0,31381	-0,30159	-0,31226	-0,30849	-0,31206	-0,30443	-0,3137	-0,31046	-0,31194
**CAM-B3LYP**	-0,26436	-0,27912	-0,27909	-0,27335	-0,28123	-0,28121	-0,26697	-0,27929	-0,27575	-0,27999	-0,26944	-0,28057	-0,27779	-0,27976
**M11**	-0,306	-0,31798	-0,31796	-0,3143	-0,31963	-0,31958	-0,30716	-0,31671	-0,31444	-0,31697	-0,30867	-0,31695	-0,31623	-0,31754
**HISSbPBE**	-0,22197	-0,23319	-0,23316	-0,22926	-0,23497	-0,23493	-0,22414	-0,233	-0,23056	-0,23351	-0,22681	-0,2343	-0,23201	-0,23341

4.2. LUMO [eV]

	6–31G(d,p)	6–31+G(d,p)	6–31++G(d,p)	6–311G(d,p)	6–311+G(d,p)	6–311++G(d,p)	cc–pVDZ	aug–cc–pVDZ	cc–pVTZ	aug–cc–pVTZ	def2–SVP	def2–SVPD	def2–TZVP	def2–TZVPD
**BLYP**	-0,06879	-0,08787	-0,08789	-0,07995	-0,09044	-0,09042	-0,07264	-0,08901	-0,08337	-0,09894	-0,07597	-0,09100	-0,08620	-0,08940
**TPSSh**	-0,06324	-0,07769	-0,07768	-0,07155	-0,07960	-0,07956	-0,06585	-0,08491	-0,07353	-0,08554	-0,07700	-0,07985	-0,08258	-0,07832
**B3LYP**	-0,05978	-0,07090	-0,07091	-0,06993	-0,07335	-0,07332	-0,05750	-0,07174	-0,06676	-0,07875	-0,06127	-0,07378	-0,06931	-0,07206
**PW6B95**	-0,05049	-0,06265	-0,06265	-0,05925	-0,06737	-0,06736	-0,05478	-0,06992	-0,05786	-0,06777	-0,05702	-0,06516	-0,06039	-0,06320
**MPWB1K**	-0,02670	-0,04099	-0,04098	-0,03437	-0,04260	-0,04257	-0,02975	-0,04100	-0,03626	-0,04368	-0,03516	-0,04313	-0,03878	-0,04224
**M06-2X**	-0,02456	-0,03618	-0,03619	-0,03315	-0,03868	-0,03865	-0,02405	-0,03630	-0,03298	-0,04386	-0,02827	-0,03795	0,00143	-0,03996
**wB97**	0,02723	0,01541	0,01539	0,01821	0,01362	0,01362	0,02904	0,01015	0,01558	0,01136	0,02485	0,01344	0,01400	0,01083
**wB97X**	0,02559	0,00908	0,00906	0,01283	0,00712	0,00714	0,01636	0,00858	0,01070	0,00342	0,01405	0,00681	0,00819	0,00490
**CAM-B3LYP**	-0,01229	-0,02607	-0,02609	-0,02298	-0,02860	-0,02858	-0,01165	-0,02683	-0,02143	-0,03256	-0,01551	-0,02862	-0,02421	-0,02715
**M11**	0,01721	-0,00097	-0,02016	0,00432	-0,00480	-0,02033	0,01514	-0,02264	0,00636	-0,02285	0,01239	0,00231	0,00404	-0,00598
**HISSbPBE**	-0,06333	-0,07460	-0,07460	-0,07198	-0,07842	-0,07678	-0,06374	-0,07474	-0,07105	-0,07861	-0,06949	-0,07676	-0,08186	-0,07524
